# A revisited history of cacao domestication in pre-Columbian times revealed by archaeogenomic approaches

**DOI:** 10.1038/s41598-024-53010-6

**Published:** 2024-03-07

**Authors:** Claire Lanaud, Hélène Vignes, José Utge, Gilles Valette, Bénédicte Rhoné, Mariella Garcia Caputi, Natalia Sofía Angarita Nieto, Olivier Fouet, Nilesh Gaikwad, Sonia Zarrillo, Terry G. Powis, Ann Cyphers, Francisco Valdez, S. Quirino Olivera Nunez, Camilla Speller, Michael Blake, Fred Jr. Valdez, Scott Raymond, Sarah M. Rowe, Guy S. Duke, Francisco Ernesto Romano, Rey Gaston Loor Solórzano, Xavier Argout

**Affiliations:** 1 CIRAD, AGAP Institut, Avenue Agropolis, F-34398 Montpellier, France; 2 AGAP Institut, Université de Montpellier, CIRAD, INRAE, Institut Agro, Montpellier, France; 3 UMR 7206 Eco-anthropologie, Département Homme et Environnement, MNHN-CNRS-Université Paris Cité, Paris, France; 4 Institut des Biomolécules Max Mousseron – (UMR IBMM), Université de Montpellier, Montpellier, France; 5 Museo Antropologico y de Arte Contemporaneo (MAAC), Guayaquil, Ecuador; 6 Museo Nacional de Colombia (MNC), Bogotá, Colombia; 7 Gaikwad Steroidomics Lab LLC, Davis, USA; 8 Department of Anthropology, University of British Columbia, Vancouver, Canada; 9 Department of Geography and Anthropology, Kennesaw State University, Kennesaw, USA; 10 Universidad Nacional Autónoma de México (UNAM), México, México; 11 Institut de Recherche pour le Développement (IRD), UMR 208 PALOC, MNHN-IRD, Paris, France; 12 Asociación para la Investigación Científica de la Amazonía de Perú (ASICAMPE), Lima, Perú; 13 The University of Texas at Austin, Austin, USA; 14 Department of Anthropology and Archaeology, University of Calgary, Calgary, Canada; 15 The University of Texas Rio Grande Valley, Edinburg, TX USA; 16 Instituto Nacional de Investigaciones Agropecuarias, (INIAP), EET Pichilingue, Quevedo, Ecuador

**Keywords:** Plant domestication, Genetics, Molecular biology

## Abstract

Humans have a long history of transporting and trading plants, contributing to the evolution of domesticated plants. *Theobroma cacao* originated in the Neotropics from South America. However, little is known about its domestication and use in these regions. In this study, ceramic residues from a large sample of pre-Columbian cultures from South and Central America were analyzed using archaeogenomic and biochemical approaches. Here we show, for the first time, the widespread use of cacao in South America out of its native Amazonian area of origin, extending back 5000 years, likely supported by cultural interactions between the Amazon and the Pacific coast. We observed that strong genetic mixing between geographically distant cacao populations occurred as early as the middle Holocene, in South America, driven by humans, favoring the adaptation of *T. cacao* to new environments. This complex history of cacao domestication is the basis of today's cacao tree populations and its knowledge can help us better manage their genetic resources.

## Introduction

The current diversity of a crop species reflects its past history where the joint impacts of environmental changes and human-plant interactions have shaped it over centuries and millennia^1^. Through human migrations and trading routes, a new diversity may emerge in a plant species resulting from exchanges, selection and genetic mixing between distant and differentiated populations. Humans have a long history of transporting and trading plants, thereby moving them out of their natural ranges and selecting varieties with traits that are of greatest interest. *Theobroma cacao* L., a greek term meaning: “the food of the gods," is one such plant that was of great economic and symbolic interest to the ancient farmers in the New World. It originated from tropical and humid regions of South America with hotspots of diversity observed in the upper Amazon basin close to the frontiers between Colombia and Ecuador^1–4^. This diversity has been classified in ten genetic groups, (Amelonado, Contamana, Criollo, Curaray, Guiana, Iquitos, Marañon, Nacional, Nanay, and Purús)^2^, and more recently classified in eleven genetics groups, with the addition of a supplementary group located in Colombia and named “Caqueta”^4^. However, it is in Mesoamerica and Central America that the ancient domestication of *T. cacao* has been particularly well documented, in part owing to the tree’s cultural significance to the ancient and current cultures of these regions. Archaeological evidence has shown its economic, social, and cultural importance in Mokaya, Olmec, and Maya populations^5–12^, and a fine flavor aromatic variety, the Criollo variety, was considered as the unique variety cultivated in Mesoamerica and Central America. Indeed, in popular culture, as for early crop scientists^13^, Mesoamerica and Central America are considered the homeland of cacao, even though it was introduced to the region through past human-mediated dispersal^7,14–17^.

Traces of the use and domestication of cacao in South America, dating back to 5300 years BP, have been documented in the Southern Ecuadorian Amazon^18^ where one of the three ancestors of another fine flavor aromatic variety, presently cultivated along the Ecuadorian Pacific coast, the Nacional variety, originated^19,20^. The modern Nacional variety is a hybrid population also involving Criollo and Amelonado ancestors, the latter of which is an old variety that was cultivated widely in Brazil during the last centuries and where it is thought to have been domesticated during the eighteenth century^21^. The presumed origin of modern Nacional variety would have resulted from a first introduction of Nacional genotypes, introduced from southeastern Ecuador to the Pacific coast, followed by the introduction, only a century ago, of Trinitario types (hybrids between Criollo and Amelonado) from Venezuela^22^.

However, many questions and uncertainties remain about the timing, way of migration and population diversity of Criollo and Nacional during the domestication steps. Moreover, no new information regarding cacao's use during pre-Columbian times has come to light so far, in South America since our previous work^18^. Our goals are now to trace the migration and use of *T. cacao* in South America from the Amazonia, its region of origin, to the Pacific coast where it was introduced and observed by the Spanish upon their arrival on the Ecuadorian coast. To this end, the presence and ancestry of *T. cacao* in ceramics from a wide range of human cultures, present in South America and spanning several millennia, from the earliest ceramic-making peoples who inhabited the Pacific coast of South America, have been studied. The analysis of ancient DNA (aDNA) from old remains of plants can be used to study and directly observe past genetic diversity of plant species of interest, thereby helping us to unravel the history of their domestication^23,24^. In this work, no archaeobotanical cacao remains could be found, but we analyzed the aDNA collected in the ceramic residues of 352 archaeological items from several pre-Columbian cultures located along the Pacific coasts of Ecuador and Colombia, as well as in Central America, and dating within the past five millennia. These residues corresponded to food residues adhered or adsorbed by the ceramic walls. The recent genetic re-sequencing of a collection of modern cacao trees representing the global diversity of the species^25^ makes it possible for us to analyze, with SNP (Single Nucleotide Polymorphism) markers, the genetic structure and relatedness between the varieties formerly consumed and the modern native populations of cacao that originated from various geographical regions. Thus, these analyses provide clues to the origin of the old varieties and the pathways of their ancient domestication. Our analysis of aDNA from various archaeological contexts, reveals a widespread dispersal and use of *T. cacao* by people in Amazonian and Pacific coast regions as well as the use of its wild relative species. In addition to recovering *T. cacao* aDNA, we also identified the presence of methylxanthine compounds, characteristic of modern *T. cacao* seeds, on ancient artifacts from several archaeological sites. Farmers currently face many threats to *T. cacao*. A better knowledge of the complex history of cacao domestication, which led to the adaptation of cocoa trees to their new environments and to their genetic mixing, at the basis of current cacao tree populations, will help to improve our breeding strategies.

## Results

**Figure 1:**
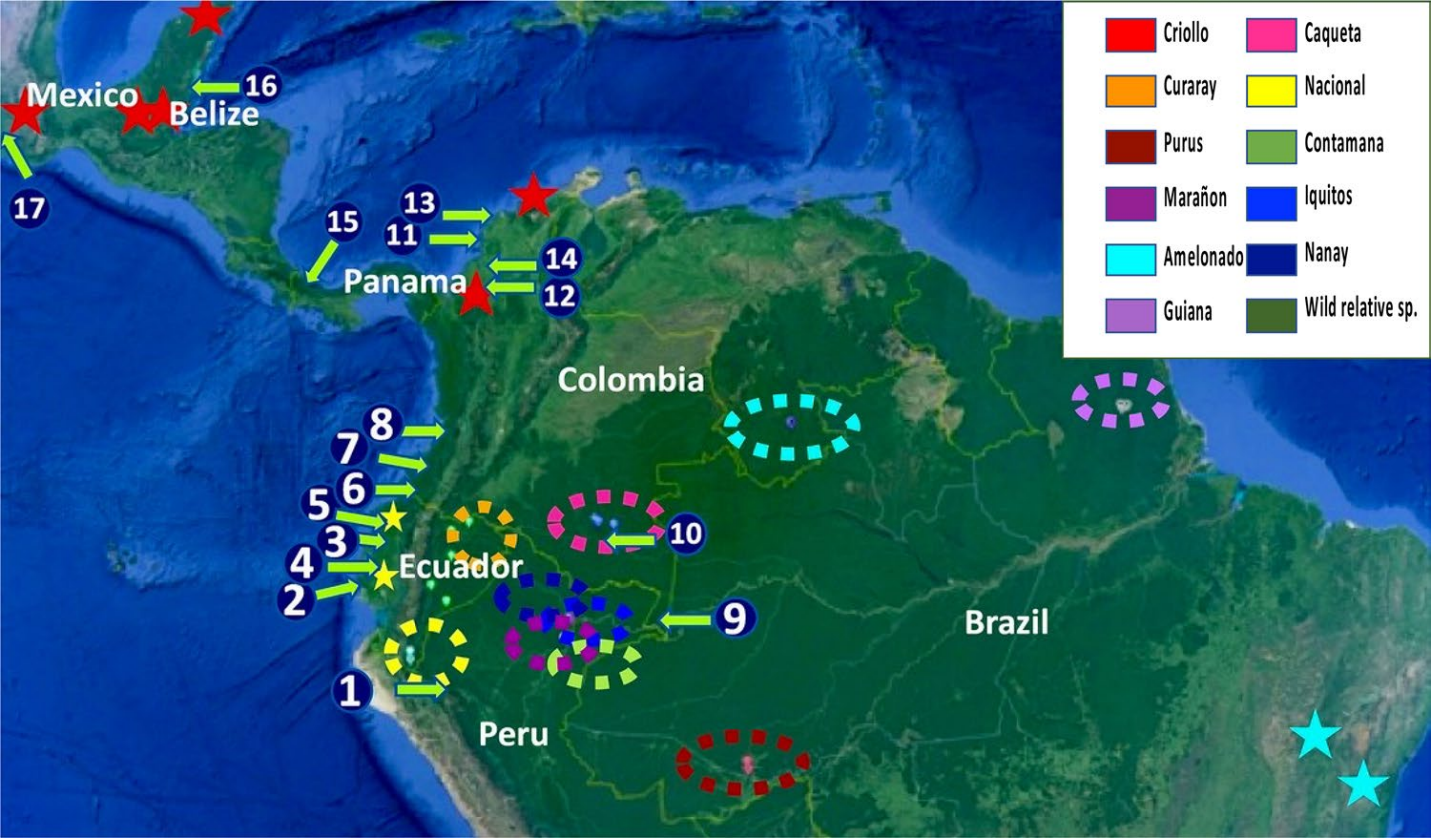
Geographical localizations of genetic groups and human pre-Columbian cultures associated to the archaeological items analyzed. The eleven genetic groups of the *T. cacao* species, as previously reported^4^, are indicated at the right top of the figure and their native areas localized in the map with the corresponding colors. Stars represent the area of cultivation of the three old varieties: Criollo (red), Amelonado (light blue) and Nacional (yellow), and with their corresponding wild genetic group, except for Criollo, for which there is no very close wild-type genetic group. The approximate sites of occupation of human pre-Columbian cultures associated to archaeological items investigated in this study are represented on the map by a number and an arrow: (1) Marañon, (2) Valdivia, (3) Machalilla, (4) Bahia/Chorrera, (5) Jama Coaque, (6) La Tolita/Atacames/Nariño, (7) San Augustin, (8) Calima Ilama, (9) Amazonas, (10) Araracuara, (11) Puerto Hormiga, (12) San Jacinto, (13) Barlovento, (14) Zenu, (15) Panama, (16), Maya, (17) Olmec.

A total of 378 ceramic residues were collected from 352 archaeological items (Table 1, Supplementary Table 1) representing 19 ancient human cultures from six countries, widespread on the Pacific coast of Ecuador and Colombia, in Amazonia, and in Central America (Fig. 1 and Table 1). Methylxanthine analyses were carried out for 326 archaeological items, and aDNA analyses were carried out for 157 archaeological items for which it was possible to construct and sequence a library. A detailed description of the archaeological items analyzed for the presence of methylxanthines or of ancient DNA of *Theobroma* or *Herrania*, a genus close to *Theobroma* and consumed by local populations, is reported in Supplementary Table 1.

**Table 1:** Number of positive archaeological items for the presence of methylxanthine and ancient DNA (aDNA) per associated cultures. The number of archaeological items for which it was possible to construct and sequence a library is indicated as “Number of archaeological items analyzed for aDNA” Methylxanthines are considered as positive with a value > 700pg/sample. Presence of *T. cacao* (or *Herrania*) sequences is considered as positive for samples showing at least five different sequences for which the first hit is *T. cacao* (or *Herrania*) after blast against the NCBI NT international database. BC: years before Christ; AD: years after Christ.

Culture	Associated cultural chronology	Total number of archaeological items analized for methylxanthines or aDNA	Source excavation (AE) or museum (MS)	Number of items analyzed for both methylxanthines and aDNA	Number of archaeological items analyzed for methylxanthines	Number of archaeological items with theobromine presence	Number of archaeological items with theophylline presence	Number of archaeological items with caffeine presence	Number of archaeologi cal items analyzed for aDNA	Number of items with *T. cacao* aDNA presence	Number of items with *Herrania* aDNA presence
Amazonas	350 BC–AD 1200	6	AE	5	6	6	0	2	5	1	0
Araracuara	AD 805–1610	13	AE	11	13	7	4	11	11	3	0
Barlovento	1560–1030 BC	6	AE	3	6	3	0	6	3	4	2
Calima-Ilama	1600 -100 BC	25	MS	12	24	14	4	20	13	12	3
Puerto Hormiga	3090–2552 BC	26	AE	12	26	20	1	26	12	10	8
San Augustin	1000 BC–AD 1	2	MS	2	2	2	1	2	2	2	1
San Jacinto	3750–2000 BC	10	AE	10	10	10	3	10	10	4	0
Zenu	200 BC–AD 1600	1	MS	1	1	1	0	1	1	1	0
Tumaco—La Tolita	700 BC–AD 400	35	AE and MS	2	35	4	0	7	2	1	1
Atacames	400 BC– AD 1530	2	MS	1	2	1	0	1	1	1	0
Bahía	500 BC–AD 650	15	MS	0	15	1	0	1	0	NA	NA
Chorrera	1000–350 BC	33	MS	8	33	5	3	3	8	7	2
Chorrera-Bahia	600–500 BC	2	MS	0	2	0	0	0	0	NA	NA
Chorrera-Jama Coaque	600–350 BC	1	MS	0	1	0	0	0	0	NA	NA
Jama Coaque	350 BC–AD 1532	26	MS	2	26	4	1	3	2	2	11
Machalilla	1600–1000 BC	6	MS	5	6	3	0	3	5	5	2
Valdivia	3900–1400 BC	96	AE and MS	41	96	35	6	27	41	19	6
Marañon	1882–1642 BC	7	AE	2	7	0	0	0	2	2	1
*Total South America*		312		117	311	116	23	123	118	73	27
Olmec	1800–1000 BC	15	AE	0	0	NA	NA	NA	15	11	1
Maya	600 BC–AD 250	20	AE	10	10	1	1	1	19	11	3
Panama	1500 BC–AD 600	5	MS	5	5	4	4	5	5	4	0
*Total Central America*		40		15	15	5	5	5	39	26	4
*Total samples*		132		133	326	121	28	129	157	99	31

### Methylxanthine analyses

*T. cacao* seeds are rich in two methylxanthines, theobromine and caffeine, and have a smaller amount of a third compound: theophylline^26^. Other South American plant species, such as mate (*Ilex paraguariensis*), Guarana (*Paullinia cupana*) and *Theobroma bicolor*^26–28^ also contain these compounds, so the methylxanthine presence in ceramic residues could be also indicative of the use of these plants in addition to *T. cacao*. However, the other species within the *Theobroma* (or *Herrania*) genus have much lower levels of theobromine and theophylline; so, the presence of high levels of these substances in the ceramics would strongly indicate the presence of *T. cacao* rather than other *Theobroma* or *Herrania* species.

**Figure 2:**
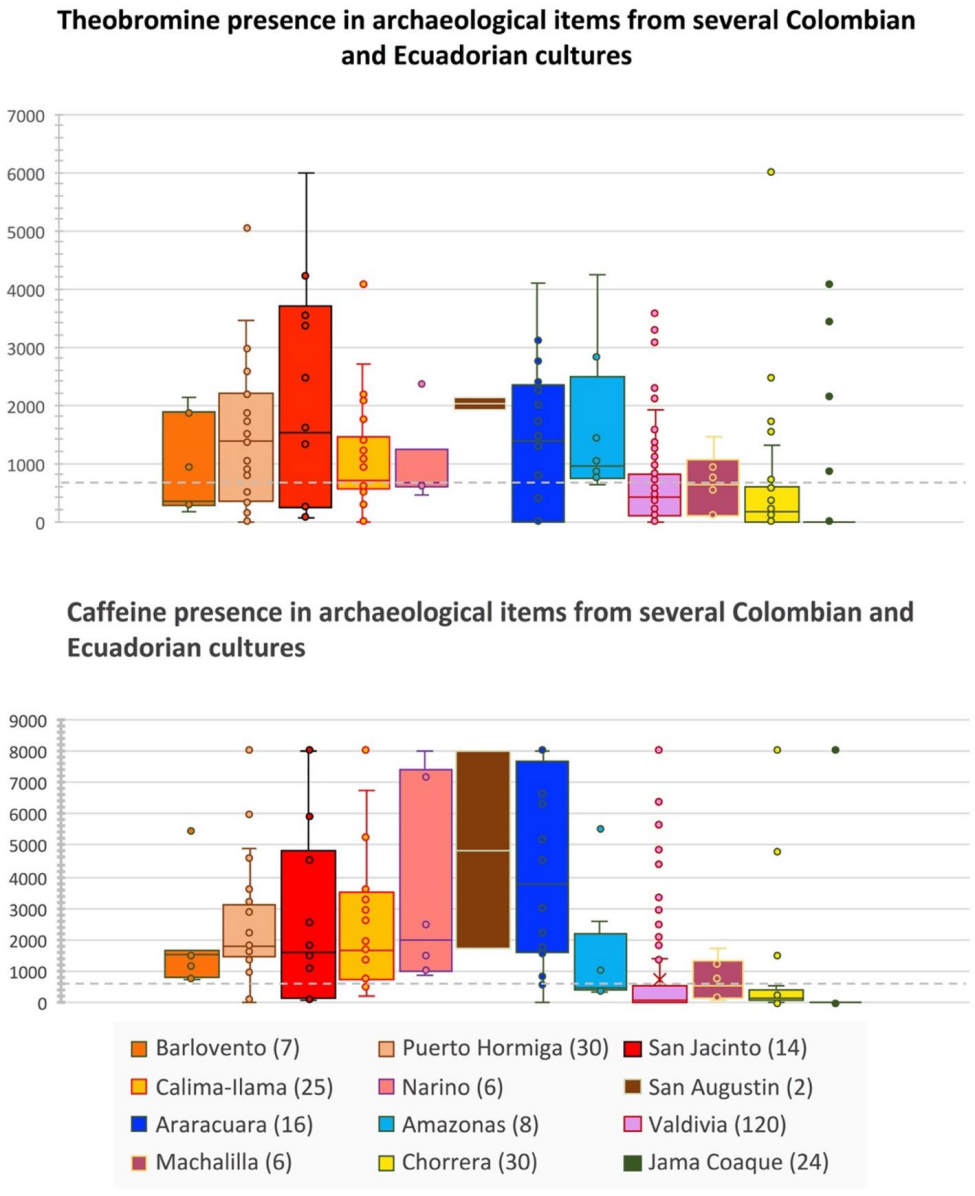
Box plots statistical representation of theobromine and caffeine values (pg/sample) observed in several Colombian and Ecuadorian cultures. The points in the graphics represent the individual values, and the numbers in parentheses correspond to the number of samples analyzed per culture. The central box is delimited by the first quartile (value below which 25% of the data falls when arranged in ascending order) and the third quartile (value below which 75% of the data arranged in ascending order lies), and with the median (middle value of the set of values with half of the values less than the median and half the values greater than the median). The ends of the whiskers are calculated using 1.5 times the interquartile space (the distance between the 1st and 3rd quartiles), and with outliers drawn over the upper limit. The dashed lines indicate the threshold at which methylxanthine values are considered positive (700 pg/sample). The number of analyzed archaeological samples per human pre-Columbian culture is indicated in parentheses.

We tested the presence of methylxanthines in the residues of 326 ceramic items, as well as with associated controls containing (positive control) or not containing (negative control) methylxanthines (Fig. 2 and Supplementary Fig. 1). Previous studies have noted that environmental contamination can occur during museum storage, and that caffeine and related methylxanthines can comprise one component of the particulate matter that can settled on artifacts in museums^29,30^. Methylxanthine contamination also may be due to human activity during excavations, and by exposure to water and/or micro-organisms^29^.

To avoid false positive results linked with possible contamination, we used the distribution of methylxanthine amounts (theobromine, theophylline and caffeine values) (supplementary Fig. 2), to define a threshold of 700pg/sample to consider a sample as positive for the methylxanthine presence (see material and methods).

Among the 311 archaeological items analyzed from South America, 116 (37%) were positive for theobromine presence, 23 (7%) for theophylline presence and 123 (39%) for caffeine presence (Table 1). Theobromine and caffeine presence were observed in archaeological items from all South American cultures except for the seven Marañon culture items (Fig. 2, Supplementary Table 2).

### Ancient DNA analyses

The ancient DNA is known to be highly degraded, characterized by scarcity and damage due to postmortem deamination^31–33^. We adapted the experimental conditions to aDNA post-mortem decay for the first steps of aDNA analyses (extraction, construction of libraries), to avoid contaminations by modern DNA (see material and methods, Laboratory environment). We analyzed 157 archaeological items for the presence of *T. cacao* or wild relatives, associated with several negative controls.

### Identification of *T. cacao*/*Herrania* specific sequences

After library construction and sequencing, 13.620 billion of useful pair sequences (with quality scores and allowing to construct a consensus sequence) were produced. Ceramic residues contain a mixture of DNA from several organisms and *T. cacao* or *Herrania* specific sequences were identified after applying two successive filters: from the whole set of sequences, a total of 618,048 sequences could first be mapped on the *T. cacao* genome (0.0045%). Then, these sequences were BLASTed on the NCBI NT international database. Among these, 19,836 sequences (3.21%) were identified as “first hit” *T. cacao* specific sequences, and 1,059 sequences (0.18%) were identified as first hit *Herrania* specific sequences (Supplementary Table 2).

**Figure 3:**
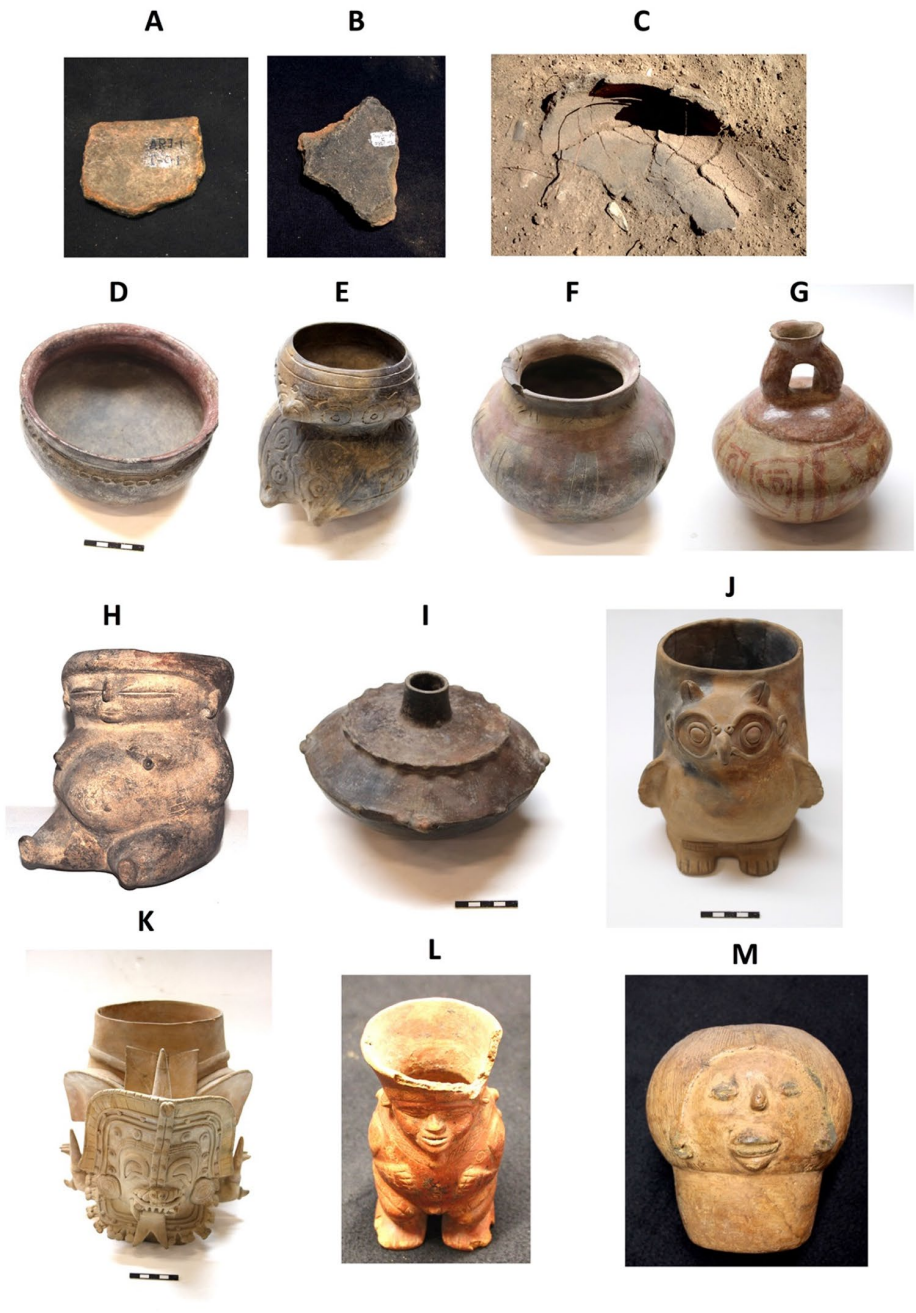
Examples of archaeological ceramic items in which we detected *T. cacao* ancient DNA presence. Item names and their associated culture: A: P236: sherd from Puerto Hormiga; B: P247: sherd from San Jacinto; C: P175: ceramic item of the Jaen Archaeological site directly collected, Marañon; D: P325: pot, Valdivia phase III; E: P345: pot, Valdivia phase II; F: P348: pot, Machalilla; G: P350: Machalilla; H: P57: effigy vessel of a pregnant woman, Chorrera; I: P137: bottle, Chorrera; J: P306: effigy vessel of an owl, Jama Coaque; K: P307: effigy vessel of mythic being, Jama Coaque; L: P213: vase, Calima Ilama; M: P214: vase, Calima Ilama.

Of the 157 samples analyzed for aDNA, 99 were positive for *T. cacao* specific sequences and 31 for *Herrania* specific sequences. Among the 118 ceramic samples from South America, 73 had *T. cacao* and 27 had *Herrania* aDNA specific sequences. As for methylxanthine presence, *T. cacao* specific sequences were observed in all South American cultures associated with ceramic items analyzed in this work (Table 1, Fig. 3).

None of our negative controls contained ancient *T. cacao* or *Herrania* DNA sequences above our threshold set at five specific sequences.

### Ancient DNA authentication

Typical postmortem DNA damages, as previously described^23^, were observed: (1) a high aDNA fragmentation for all analyzed samples whose mean length of aDNA fragments is 81,65 bp (Supplementary Fig. 3); (2) a decreased PCR (polymerase chain reaction) amplification intensity when increasing the length of amplified DNA fragments as observed in all samples positive for cacao presence and reported in Supplementary Fig. 3 for a part of them; and (3) other postmortem damages, typical of aDNA were also observed, as an enrichment of purines (A and G) and a higher C– > T substitutions around the ends of aDNA fragments, due to cytosine deamination, as represented in Supplementary Fig. 4 for two samples.

### Relationships between aDNA and methylxanthine presences

The presence of theobromine was mostly our first selection criterion to select the samples to be analyzed for aDNA. We focused on the 120 archaeological items analyzed for both methylxanthines and ancient DNA, and positive for at least one of them, as reported in Supplementary Table 2. Among them, 96 items are positive for theobromine presence, regardless of the presence or not of caffeine or theophylline. Among these 96 items, 62 (65%) were positive for *T. cacao* aDNA presence. If we consider the 26 items positive for the presence of theophylline, 21 of them (81%) contain also *T. cacao* aDNA. Similarly, in the same set of 120 archaeological items, 81 items are positive for caffeine presence, and 62 of them (76%) are also positive for *T. cacao* aDNA presence.

Thus, a high percentage of *T. cacao* presence can be predicted by the analysis of either of these methylxanthine measurements despite their presence in several other South American plants. Among the same set of 120 archaeological items, if we consider only the 85 items positive for cacao aDNA presence, 62 of them (73%) are positive for theobromine, 62 (73%) are positive for caffeine and 21 (25%) are positive for theophylline. The lower success rate for the latter is probably explained by the much lower theophylline levels (5000 to 10,000 times less) existing in cacao beans^26^, making it a less sensitive predictor for the presence of *T. cacao* in the ceramics*.*

### Diversity and ancestries of cacao ancient DNA

To identify the genetic origins of the *T. cacao* aDNA present in the different archaeological cultures studied here, we tried to elucidate their genetic ancestry and to link them to possible routes of introduction of *T. cacao* from Amazonia. To this end, we selected a reference set of 76 modern accessions, recently re-sequenced^25^, representative of the diversity of the *T. cacao* species. We established a phylogenetic tree with 460 SNPs (Supplementary Fig. 5), confirming previous results that show *T. cacao's* structure in eleven genetic groups^4^. This phylogenetic tree highlights and confirms the closest genetic distance between Criollo and the new Caqueta genetic group located in Southern Colombia (Fig. 1), a region geographically close to the Araracuara archaeological site^34^. It also confirms the close relationships between some modern Nacional ancestors^19^ and genotypes of the Nacional group located close to the Palanda archaeological site (PAL) in the southeastern Ecuador.

The phylogenetic tree also confirms the low genetic distance between all the Guyanese and Peruvian genetic groups—Marañon, Guiana, Iquitos, Nanay, Amelonado^2,4^. The Curaray group appears close to the Criollo group in accordance with previous results^2^. This phylogenetic tree also displays a close genetic distance between the Nacional and Contamana groups contrary to previous results obtained with microsatellite markers^4^ or gene presence and absence variations (PAV) markers^25^. Overall, these 76 accessions provide a good representation of the currently known *T. cacao* diversity and genetic groups, making them suitable candidates to analyse the ancestry of ancient DNA sequences. We also added five representatives of wild relative species to our analysis, to identify the possible consumption, in ancient times, of species close to *T. cacao*.

As reported in Table 1, 157 archaeological items could be analyzed for the presence of *T. cacao/Herrania* ancient DNA (independently of methylxanthines analyses). Among them, 99 contained aDNA from *T. cacao*. After SNP extraction of these sequences, common to both the reference collection and each of the archaeological samples, structure genetic analyses could be carried out on 61 archaeological items and genetic distance analyses carried out on 66 of them.

**Figure 4:**
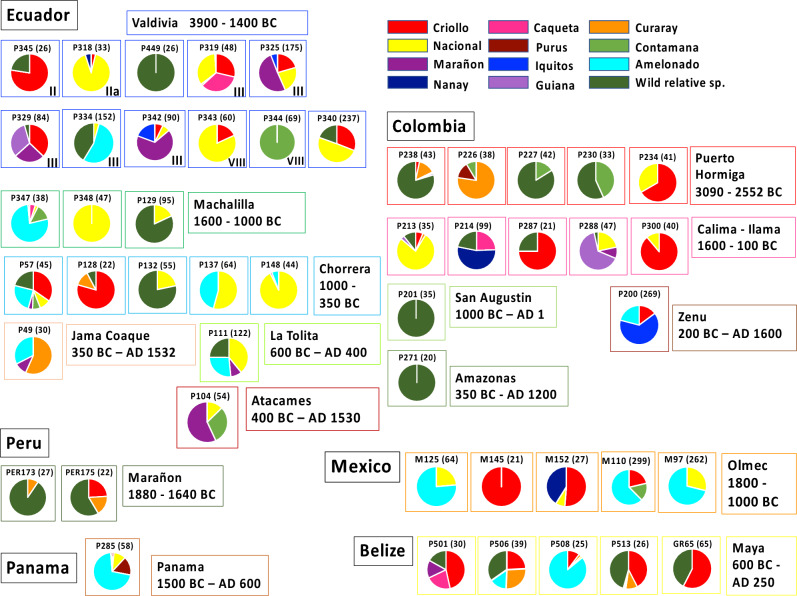
Visualization of the genotype membership proportions of *T. cacao* genetic groups and wild relatives for each archaeological item. The genetic structure of each archaeological item residues was analyzed individually with the reference collection. The Valdivia phase is indicated at the right and bottom side for each Valdivia sample when determined. The structure of *T. cacao* ancient DNA sequences from ceramic residues was determined using a Bayesian model-based clustering method, implemented in the STRUCTURE software V2.3.4^35^. The genotype membership proportion was calculated using a set of at least 20 SNP markers, common to the sample and to the reference collection and is indicated by colours corresponding to the genetic groups mentioned at the top right side of the figure. Only archaeological items with unambiguous ancestry values between two groups are reported in this figure. The complete ancestry values are reported for all analyzed items in Supplementary Table 3. The number of SNP used for each Structure analysis is indicated in parentheses.

All detailed geographic origin information for these samples is gathered in Supplementary Table 1. A variable number of SNPs common to the aDNA and the reference collection (Supplementary Table 3) can be observed, but all items had a minimum of 20 common SNP markers. Each aDNA sample was analyzed individually with the reference collection using their common set of SNP markers. We first analyzed the structure of the samples and estimated the genotype membership proportions of *T. cacao* genetic groups and wild relatives for each archaeological item, using Structure software^35^ (Supplementary Table 3, Supplementary Fig. 6 and Fig. 4). We identified a high degree of diversity of cacao ancestries among the human cultural groups. In the Valdivia culture, we found samples related to the Marañon and Contamana genetic groups, suggesting interactions with the Peruvian region where these *T. cacao* groups originated, but also samples related to Nacional, Criollo and Amelonado groups. Plots structure, at different values of K is reported as examples for two Valdivia items, having important Amalonado, Criollo and Nacional ancestries, confirming these ancestries at different values of K (Supplementary Fig. 7). A similar diversity of origins was found in more recent Ecuadorian cultures such as Machalilla and Chorrera, with cacao samples displaying genetic structures that are either completely Nacional, or hybrids with Nacional mixed with other types, including Amelonado, as is observed presently in the modern Nacional variety. The samples from the Marañon culture in Amazonian Perú, show the presence of Criollo and Curaray genetic groups.

A similar range of cacao genetic diversity can also be observed in ancient cacao samples from Colombia, with some samples highly related to Criollo observed in samples from Puerto Hormiga and Calima-Ilama cultures. Likewise, we observed diverse genetic origins of ancient cacao samples from Olmec and Maya sites in Central America. We found that, contrary to previous research, Criollo was not the only variety consumed. Instead, we found evidence that Amelonado, Nacional and/or Iquitos genetic groups were present by the Olmec period. The Nei genetic distances calculated between the ancient DNA sequences and the modern genetic groups confirmed the results of structure analyses (Supplementary Table 4).

We also found that wild species of *Theobroma* or *Herrania* genus were consumed by nearly all cultures that we analyzed. In a few cases, such as some samples from the Barlovento, Chorrera, and Leticia sites, it was possible to identify a *Herrania* species used by human populations. *Theobroma speciosa* was also identified in a sample from a Calima Ilama item.

## Discussion

Recent findings document the domestication of *T. cacao* in the Ecuadorian Amazon region, its region of origin, by at least 5300 years ago^18^. Our new findings demonstrate the large landscape of domestication of cacao, out of its area of origin, along the Pacific coast of South America, occurring concurrently during this same early time period and in subsequent time periods. These new findings are revealed by the presence of *T. cacao* ancient DNA on almost 30% of the ceramic items that we tested, that were used in both domestic and ritual activities. The wide range of ceramic artifacts containing evidence of ancient *T. cacao* DNA, demonstrates the extensive and ongoing use of both *T. cacao* and its wild relatives by ancient peoples who lived along the Pacific coast of northern South America.

*Theobroma cacao* originated in Amazonia and therefore its presence along the Pacific coast, reveals past interactions between Amazonian peoples and their neighbors to the west along the coast. These interactions may have included both human migration and trade exchanges that encouraged the dispersal of cultivated plants along with a host of other trade items^36^. Several authors have reported intense commercial exchanges among Amazonian regions^37,38^ all along the vast river networks throughout Amazonia. Goods, plants, and people could have travelled up to 1000 km, exchanging complementary tools, foods and other materials needed for people’s livelihoods, as well as knowledge and ideas that were an essential part of Amazonian cosmology^39,40^. Amazonia was a major world center of plant domestication, where selection began in the Late Pleistocene to Early Holocene^41,42^, thus, exploiting and generating a new diversity provided by a genetic mixing of introduced *T. cacao* trees from different origins. Within Amazonia, an important center of resources was reported in the Iquitos region of Perú^41^, where several *T. cacao* genetic groups originated: most notably the Marañon, Nanay, Iquitos and Contamana groups. In Colombia, an independent center of domestication was reported^43,44^, and a study of plant food production in Colombian tropical forests reported the adoption of exogenous plants domesticates, as manioc and maize, as early as the middle Holocene^45^. Interactions between Amazonia and the Pacific coastal peoples that involved the use and domestication of *T. cacao* likely occurred during the earliest stages of agriculture. This inference is based on our observation that cacao originating from several *T. cacao* genetic groups located in the Peruvian Amazonia, was observed in the oldest Pacific coast cultures of Valdivia, in Ecuador, and Puerto Hormiga and San Jacinto in Colombia, dating to more than 5,000 years ago. In samples from the Valdivia culture sites, dating to Phase III (2950-2600 BC), the presence of *T. cacao* genotypes originating from the Peruvian Marañon and Nanay groups suggests that people in this region had early long-standing contacts with the Peruvian Amazon. Genotypes related to the Nacional group were also observed in the Valdivia ceramic residues. This genetic group is located in southeastern Ecuador, where the Mayo-Chinchipe-Marañon culture existed contemporaneously with the coastal Valdivia culture. Mobility was one of the characteristics of the Mayo Chinchipe-Marañon people. They navigated the many riverine tributaries that flow into the main channel of the Amazon River, thus allowing the rapid and extensive spread of plants (including *T. cacao)* and other products, throughout this vast region^46,47^. A similar situation is encountered for the samples originated from the Caribbean coast of Colombia (Puerto Hormiga and San Jacinto) where cacao genotypes related to the Marañon, Contamana and Iquitos genetic groups, originating in Perú, were observed, reflecting direct or indirect early contacts with the Peruvian Amazon. Even if modern-day diversity has changed over millennia, the concordant ancestry and parentage results obtained by analyses of genetic structures and genetic distances support the probable origin we identified and the genetic mixing of cacao trees introduced to the Pacific coast.

*Theobroma cacao* was also introduced to Central America, but its introduction from the Amazon, either northward overland, or by sea along the Pacific coast, still raises many questions. Several authors have suggested exchanges between the Pacific coast of Ecuador and Mesoamerica, based on many similarities between ceramics of the Pacific coast of Ecuador and the Pacific coast of Guatemala^37,48–50^. More recently the evidence for pre-Columbian maritime long-distance contacts between western Mexico and the Pacific coast of northwest South America were synthesized and reassessed^51^, considering an area spanning 4000 km of coastline and over 4000 years of interaction. Early contacts could be highlighted: the observation of a ceramic motif of the early formative Valdivia VI phase (2200-2000 BC) during the Mesoamerican Early Formative Period^52^, evinces early long-distance Pacific coastal interactions. Several other facts have been pointed out^17^ showing that maritime navigation was possible at early times and could have supported cacao’s dispersal from Ecuador to Mesoamerica through vast interconnected political-economic networks. Recent findings based on ancient DNA analyses have shown an early dispersal of maize, first domesticated in Mexico, to Perú 6700–5000 cal years BP (Before Present), through a rapid coastal migration route from the Pacific lowlands^53^. These findings demonstrate the early and possible rapid exchange of plants between Mesoamerica and the Pacific coast of South America where the Pacific coast of Ecuador and cacao may also have been involved in these exchanges.

Our archaeogenomic results have shown the diversity of the genetic origins of the cacao varieties consumed by ancient peoples, and call into question the previously proposed patterns of the introduction of cacao trees on the Pacific coast of Ecuador and in Central America. The three ancestors of the modern Nacional variety—Criollo, Amelonado, Nacional—already existed on the Pacific coast during Valdivia times, contrary to what we had previously thought. The present study also suggests that the domestication of the other fine flavor variety, the Criollo variety, is likely to be older than previously thought. While Criollo’s origin remains unknown, it shows a greater proximity to the Caqueta genetic group, located in Colombian Amazonia^4^. Often hybridized with other genetic groups, it is observed in archaeological samples from some of the oldest cultures in Ecuador (Valdivia) and Colombia (Puerto Hormiga, Calima Ilama). Criollo is characterized by many private alleles^4^, suggesting that the Criollo group probably results from a long domestication process, for which a loss of genes has been recently characterized by a pangenome study showing that Criollo has the fewest genes compared to other genetic groups^25^. Criollo was supposed to be the unique variety cultivated in Central America during the Olmec and Maya periods^7,14,15^. However, our results indicate that this was not the case. Criollo, along with other genetic groups from different regions were all present in ancient Central American cultures. Among these, the main ones were the Nacional and Amelonado genetic groups, respectively originating from southeastern Ecuador, and northern Brazil.

These results have shown the complex and early history of cacao domestication and suggest that it was linked to long-distance trade and exchange patterns that started at least at mid Holocene times. They also demonstrate the effectiveness of archaeogenomic approaches in tracing plant domestication histories. Ultimately, cacao's long history is intimately intertwined with the diversity of new geographic environments and cultural groups where it has thrived and evolved, with intense gene-flows between remote *T. cacao* populations and the emergence of hybrid forms, favorable to their adaptation to new environments and adoption by local human cultures.

The multi-billion dollars cacao industry supports the economy of many countries and cacao is one of the world's most important cash crops. However, *T. cacao* is subject to many threats, including the susceptibility to disease and climate change. Our multidisciplinary approach, involving archaeogenomics, genomics, archeology and biochemistry is a key approach to unraveling the complex ancestry of *T. cacao* underlying today's cacao populations. It will help to better manage and exploit genetic resources in order to deal with these threats.

## Material and methods

### Cultural provenance of the archaeological items analyzed

Residues from the interior walls of ceramics were collected from archaeological items stored in museums or coming from direct excavations. Residues were collected from a total of 352 items, from 19 pre-Columbian cultures widespread in South America (Ecuador, Colombia, Perú), and in Central America (México, Belize, Panama). These cultures span about 5000 years and are mostly scattered along the Pacific coast. (Fig. 1 and Supplementary Table 1).

### Collection of ceramic residues

No conserved cacao organs or cacao seed fragments were found on the ceramics. Only traces of putative charred food residues adhering to the interior of the ceramics walls or sherds could be collected.

Most of the collection of ceramic food residues were made using rayon swabs (Copan-Italia) saturated with extraction buffer (0.1 M Tris, 0.45 M EDTA and 0.25 mg/ml proteinase K)^54^. The swabs were wiped against the inner walls of the ceramics and stored in their sterile box at 4°C until returning to the CIRAD-AGAP laboratory (Montpellier-France), where all samples were stored at -20°C. About 10 swabs were collected per ceramic item. For the Maya and Olmec samples, the interior surface of each ceramic vessel or sherd was lightly scraped using a new piece of fine-grained sandpaper, and the powder conserved for further analyses.

### Methylxanthine analyses

Three methylxanthine components present in *T. cacao* were analyzed: theobromine, theophylline and caffeine. Either one rayon swab or 0.5 g of powder was used for analyses of the archaeological items.

#### Extraction procedure and biochemical analyses

The analyses were made in two different laboratories:

1. The laboratory of biochemistry from Gaikwad Steroidomics Lab LLC, Davis (USA).
  Extraction procedure and biochemical analyses made in this lab were carried out as previously described^18^.
2. The « laboratoire de mesures physiques », Institut des biomolécules Max Mousseron, Université de Montpellier (France).

Extraction procedure was slightly different: Samples were incubated with 600 μl milli-Q water at pH3, at 80 °C for 30 min. After incubation, samples were vortexed and centrifuged. the supernatant was transferred in a 35 µm Macherey filter column and centrifuged at 10,000 g for 10 mn. The supernatant was desiccated by speedvac, a vacuum concentration system, and then diluted in 10 µl of milli-Q water.

*UPLC-MS/MS* (Ultra-Performance Liquid Chromatography—Mass Spectrometry) analyses were carried out according to the following protocol:

Theobromine, theobromine-d6, theophylline and caffeine reference standards were purchased from Sigma-Aldrich. Acetonitrile, methanol and formic acid were ULC/MS grade and the water was milli-Q grade. The analyses were recorded on a LC–MS 8050 triple quadrupole (Shimadzu) coupled to a Nexera chromatographic chain (Shimadzu). Positive mode electrospray ionization was used, with interface voltage at 4kV, nebulizing gas flow of 3L/min, heating gas flow of 10 L/min, interface temperature of 300 °C, a DL temperature of 250 °C, a heat block temperature of 400°C and a drying gas flow of 10 L/min. The transitions and the collision energies were optimized for each compound: 181.10 → 138.20 with collision energy of -18 eV for theobromine, 181.10 → 124.15 with collision energy of -18 eV for theophylline, 195.00 → 138.20 with collision energy of -20 eV for caffeine and 187.20 → 144.15 with collision energy of -18 eV for theobromine-d6.

Before the analyses in LCMS, the samples were passed over C18 ODS 100mg Solid Phase Extraction (SPE) (Agilent). For this, the columns were conditioned with 1 mL of methanol, followed by 1 mL of water. The samples were taken up in 100 µL of water acidified with 1% formic acid before being placed on the columns.

Then 1 ml of water was passed through. The products of interest were unhooked from the column by passing 1 ml of methanol followed by 0.5 ml of isopropanol. For each sample, these two solvents were combined and dried. The samples were taken up in 20 µl of solution of theobromine-d6 at 5 ng/mL and 10 µl were injected during the LCMS analysis.

Analytical separations on the UPLC system were conducted using an Acquity UPLC BEH C18 1.7 μm column (50 × 2;1 mm) at a flow rate of 0.6 ml/min. The temperature of the column oven was 40°C. The gradient was started with 100% A (0.1% formic acid in H_2_O) and 0% B (0.1% formic acid in CH_3_CN). Then the percentage of eluent B was increased linearly to be at 14% at 0.7 min. We remained on this plateau until 1.25 min, before increasing the percentage of B linearly to 50% at 1.5 min, then to 100% B at 3 min. The run time was 5 min. The data obtained after LCMS coupling were processed by the Labsolution Insight software (Shimadzu).

For each compound, standard solutions at 1mg/ml were prepared. Theobromine-d6 was used as an internal standard. From these stock solutions, mixtures grouping the compounds theobromine, theophylline, and caffeine at different concentrations (1; 2; 4; 6; 8; 10 and 15 ng/ml) and theobromine-d6 at a concentration of 5 ng/ml were used as calibration solutions. Two other mixtures of compounds were made from new weightings to create quality control (QC) solutions at concentrations of 1.5 and 9 ng/ml. The QCs were injected with the standards to validate the calibration line. A solution consisting only of the internal standard was used as a blank as well as negative controls for which rayon swabs, not wiped against the inner walls of the ceramics, were analyzed following the same protocol of extraction and UPLC-MS/MS analyses. The retention times obtained were 1.05 min for theobromine and theobromine-d6, 1.38 min for theophylline and 1.70 min for caffeine. From the concentrations obtained and the sample recovery volume, the quantities in pg of the various compounds were calculated.

#### Determination of methylxanthine threshold

Ideally, the threshold for a positive presence of methylxanthines depends on the Limit of Detection (LOD), where we can observe a signal for the analyte of interest, but too small to be measured accurately, and on the Limit of Quantitation (LOQ) of the analyte when no signal is observed. This is determined by the “signal” to “noise” ratio. However, possible airborne contamination during storage of archaeological items in the museum reserves was already reported^28,29^ and could increase the values. To prevent false positive results, we established methylxanthine values distribution (Supplementary Fig. 2) to estimate the possible environmental background linked to these possible levels of contamination and establish a threshold for the detection of positive samples. After plotting the distribution of methylxanthine amounts (theobromine, theophylline and caffeine values) observed in all samples (Supplementary Fig. 2), we observed a clear break in these distributions around 200 pg/sample for each series of methylxanthine values. The high number of values < 200 pg/sample may correspond to the ambient contamination already described^29^. Thus, to be conservative we chose a threshold of 700 pg/sample and considered a sample as positive where the methylxanthine amount was > 700 pg. All values < 700 pg were considered and reported as “0” in Supplementary Table 2.

We also ran positive and negative controls (Supplementary Fig. 1). In these analyses all negative controls were < 250 pg/sample.

### Laboratory environment for ancient DNA analyses

The ancient DNA is highly degraded, characterized by scarcity and damage due to post-mortem decay and deamination^31^. We adapted the experimental conditions to prevent contamination by modern DNA, preferentially amplified during PCR steps: all pre-PCR experiments were conducted under sterile conditions in the platform “Paléogénomique et génétique moléculaire” (P2GM) of the French “Muséum National d’Histoire Naturelle” at the “Musée de l’Homme” (Paris). This laboratory is dedicated to ancient DNA analyses mainly conducted on humans and animals and there has never been previous work on *Theobroma* species. It is equipped with positive high-pressure air system, with continuous filtering of incoming air, daily UV light irradiation, laminar flow hoods with HEPA filters, and all surfaces frequently cleaned. The experimenters wore whole-body protective clothing including gloves and shoe protection.

### Extraction of ancient DNA

Ancient DNA extraction from ceramic wall was first carried out on amphoras^55^. The protocol used to extract *T. cacao* ancient DNA from ceramic items collected in the South Amazonian region^18^, were adapted in this work as follow:

Four swabs were used for each aDNA extraction made from ceramic residues. Ancient DNA extraction was made with the Qiagen, DNeasy PowerLyzer PowerSoil Kit, which effectively removes PCR inhibitors such as humic acids, and according to the described manufacturer’s procedure except for the binding step for which the concentration of the C4 saline solution was increased 1.6 times to retain the smallest fragments. One or two independent extractions (made for 30 archaeological samples) were made for each archaeological sample, named MX.

Several negative controls were processed alongside the ancient samples.

### Libraries construction

Construction of the libraries using the NEXTflexTM Rapid DNA-Sequencing Kit (Bioo Scientific) were carried out for MX1 to MX12 according to the protocol indicated in the kit.

Dual-indexed Illumina sequencing libraries were prepared for MX13 to MX232 using three main reactions^56^: blunt end-repair, adapter ligation, and nick fill-in reaction as reported in supplementary methods.

### Sequencing steps

All samples were sequenced in paired ends (2 × 150 pb) on the iGenSeq core facility of ICM (Institut du Cerveau et de la Moelle Epinière—Paris). Each run was performed on ILLUMINA NOVASEQ 6000 with 300 cycles cartridge (150PE). Cartridge was choose depending of the number of samples to obtain at least 2*30 Millions of 150 bases reads per sample. Bcl files were converted in fastq format with ILLUMINA bcl2fastq.

At the beginning of the experiments, additional sequencing of aDNA samples (MX1 to MX12) were made on the Montpellier GenomiX (MGX) platform (Montpellier-France).

The aDNA libraries were sequenced according two possible different strategies reported for each sample in Supplementary Table 1: or directly by whole genome sequencing (WGS) or after a step of Targeted capture (TC) as described previously^18^. The targeted captures were carried out with a custom-designed Mybaits sequence capture kit (V 3.02) provided by the Microarray company and defined from 4847 unique nuclear *Theobroma cacao* sequences containing SNP sites. These SNP were identified by GBS (genotyping by sequencing) on a collection of *Theobroma* and *Herrania* genetic resources, and are located in genes for about 70% of them. The pipeline used for filtering, demultiplexing, and processing the reads is already described^25^.

### Bioinformatic sequences treatment

The food ceramic residues contain ancient DNA from a mixture of several species of bacteria, fungi, plants, and animals, and often with many similarities between sequences from different species. Thus, filters were necessary to specifically identify the *T. cacao* sequences. A first filter was the mapping against the *T. cacao* genome and a second filter was necessary to identify the sequences specific to *T. cacao* through a BLAST against data sequences from the NT NCBI international database.

To apply such a selection, sequencing adapters and low quality nucleotides (quality value < 20) were first removed from Illumina paired-end reads using Cutadapt v3.4^57^ and Trim Galore v 0.6.6. Reads shorter than 30 nucleotides after trimming were discarded. Then, pairs of reads were merged with FLASH v1.2.11^58^, using default settings, and redundancy from the merged DNA fragments was reduced using cd-hit-dup program (parameter -e 0) from the CD-HIT package v.4.8.1^59^. Then, low complexity/entropy sequences were removed from the pre-selected aDNA fragments using bbduk program (parameter entropy = 0.7) from BBTools v38.90^60^. Resulting sequences were then aligned to the *Theobroma cacao* reference genome^61^ with bowtie2 v2.4.2^62^ parameter –very-sensitive) and sequences that aligned at least once to the cacao genome were conserved for further analysis. Remaining sequences were then searched against the GenBank nucleotide collection NT with NCBI BLAST + v2.10.1^63^. Blast results were passed to the BASTA^64^ taxonomic classification tool v1.3.2.3 and only sequences with the best Blast Hit being *Theobroma cacao* or *Herrania* were kept for further analysis. However, these filters are very stringent, as only sequences appearing as 1st hit *T. cacao* were conserved, excluding any *T. cacao* DNA sequences homologous to other species after blast on international databases.

The presence of *T. cacao* (or *Herrania*) sequence was considered as positive in an archaeological sample when at least five different *T. cacao* (or *Herrania*) first hit sequences were identified in the sample. None of our negative control had ancient *T. cacao* or *Herrania* DNA sequences above this set threshold. We used Krona schemes to visualize the relative abundances of the several sequences species within the metagenomic classifications, identified as “first hit” after blast against the NCBI NT database^65^, as represented in Supplementary Fig. 8 for two archaeological items from the Valdivia and Puerto Hormiga cultures.

### Ancient DNA authentication

To further support the antiquity of the DNA extracted from ceramic residues, typical signatures of post-mortem DNA damage^23^, such as small size fragments and chemical damages particularly present at the ends of aDNA fragments were searched for. Pre-selected ancient DNA fragments, obtained after aligning the reads on the *T. cacao* genome were processed using MapDamage V2.2.1^66^. Several DNA damages could be analyzed:

#### Mis-incorporations observed at the ends of aDNA fragments

An enhanced cytosine deamination observed mostly in 5’ overhanging single strand DNA ends will translate itself into an excess of cytosine to thymine (C to T) mis-incorporations at 5’ ends of aDNA sequences (and complementary guanine to adenine (G to A) at 3’ ends when PCR amplified. MapDamage permits evaluation of these transitions in comparison with the corresponding *T. cacao* genome sequences.

#### Purine frequency around the ends of aDNA fragments

Purine (A and G) deamination can result in an enhanced fragmentation of DNA^23,67,68^ and MapDamage allowed evaluation of the purine frequency around the ends of aDNA fragments.

#### Evaluation of DNA fragmentation due to post-mortem damages

Mostly small DNA fragments remain and can be sequenced because of post-mortem depurination^67,68^, leading to DNA strand fragmentation. We used MapDamage V2.2.1.^66^ to display the fragment size distribution as reported in Supplementary Fig. 3 for all positive samples.

#### Impact of amplified aDNA fragment size on PCR intensity

In the case of ancient DNA, PCR amplifications with primers amplifying fragments of an increased length will be effective with a decreased intensity depending on the length of the DNA fragments^69^.

Primers were designed in the mitochondrial *Cytochrome C oxidase* subunit 2 gene, amplifying DNA fragments differing in length but having a similar PCR efficiency tested on a modern DNA (Criollo B97-61 genotype) (Supplementary Table 6): Cyto 66b: 66 bp, Mito-197: 197 bp, Mito-290: 290 bp, Mito-543: 543 bp.

Real-time PCR analysis was carried out in a BioRad CFX96 Touch Real-Time PCR Detection System (Bio-Rad Laboratoires) using the following steps: 98°C for 3 min; a touchdown PCR [initial 10 cycles; 98°C for 15 s, 58°C for 20s (-1°C every cycle), 72°C for 20s], followed by 50 cycles of 98°C for 15°C, 48°C for 20 s, 72°C for 20s, then a final step at 72°C for 8 min. To confirm product specificity a melting curve analysis was performed as the last step. The real-time PCR was carried out in a 10 μl reaction volume containing 5 μl of 2X « SsoFast EvaGreen Supermix » (Bio-Rad Laboratoires), 0.1 mM of BSA (ref. B9200, New England BioLabs), 500 nM of forward and reverse primers (Supplementary Table 6), 1 µl of water, and 2 µl of DNA extracts. In each run, blank (negative control) and a positive control were added.

Data were analyzed using CFX Maestro Software (Bio-Rad Laboratoires) set with default parameters to determine the cycle threshold (*C*T), *i*.*e*. the number of PCR cycles required for the fluorescent signal to exceed the background level.

### SNP identification

A re-sequencing and pan-genome project of 216 modern *T. cacao* accessions was recently carried out^25^. The genomes of 185 modern accessions of *T. cacao* and five accessions of cacao wild relative species (*T. grandiflorum*, *T. bicolor*, *T. speciosa* and *Herrania nitida*) were newly sequenced using the whole genome shotgun Illumina technology (NCBI Bioproject PRJNA558793). The generated 150 bp paired-end reads were mapped on the V2 cacao reference genome^61^ using Bowtie2 (v. 2.4.2)^62^. SNPs were then called using NGSEP V. 4.0.0^70^, using filtering criteria based on genotyping quality (-q 40) and on a minimum read death to keep a genotype call (minRD 5–10) and a SNP database was established comprising 31,910,149 SNPs. A subset of 76 *T. cacao* accessions representative of the genetic diversity of the species and five relative wild species was selected to perform genetic analyses aiming to identify the ancestry of each archaeological sample residue.

To take into account the potential deaminated DNA damage at the ends of the fragments, we followed the suggestion to remove the SNP, corresponding to potential C-to-T/G-to-A substitutions, located at the two first bases of each end of aDNA fragments up to a percentage of 2 to 4% C-to-T substitutions observed at the ends of aDNA fragments^71^. In our case, using Mapdamage software, a mean of 4 to 5,5% of C-to-T substitutions were observed at the ends of aDNA fragments. So, to reduce the risk of false SNP, we remove from the analyses the putative SNP located at the first five bases of each end of aDNA fragments, and corresponding to potential C-to-T/G-to-A substitutions only.

To determine the SNP alleles within the ancient DNA fragments, the genomic regions of modern accessions, homologous to the aDNA sequences, were identified using Blastn, and their SNP extracted, given the known position, on the cacao genome, of SNP identified in the modern accessions: the aDNA sequences were aligned with the homologous modern sequence, and its corresponding SNP alleles extracted. Several SNPs could be identified in a given aDNA sequence.

Only ancient DNA sequences mapping in a unique location on the cacao reference genome were retained for further genetic analyses. About 3% of ancient DNA fragments for which the SNP allele does not correspond to any of the two alleles present in the collection of modern accessions, which could be explained by substitutions due to aDNA degradation, were discarded. Between 20 and 528 SNPs were identified according to the aDNA samples, with a mean of 74 SNPs per Sample.

### Reference collection

To identify the genetic origin of ancient cacao DNA collected in the ceramic vessels, a reference collection was selected. This collection includes 76 *T. cacao* accessions representing the 11 genetic groups recently identified^4^: Criollo (8 ind), Caqueta (8 ind), Curaray (8 ind), Nacional (8 ind), Purus (7 ind), Contamana (4 ind), Marañon (7 ind), Iquitos (7 ind), Amelonado (4 ind), Nanay (8 ind), Guiana (7 ind).

This collection includes also five accessions from four wild relative species: *Herrania nitida* (2 ind),* Theobroma grandiflora* (1 ind)*, Theobroma bicolor* (1 ind)*, and Theobroma speciosum* (1 ind)*,* (Supplementary Table 5 and Fig. 1).

A phylogenetic tree was constructed with the Darwin software^72^ for the 76 *T. cacao* accessions constituting the reference collection and with a set of 460 SNP markers selected among the SNPs identified in the aDNA sequences, and widespread in all chromosomes. A distance model, based on the dissimilarity matrix calculated using the neighbor joining assembly method^73^, with 500 bootstraps as implemented in Darwin 6.0.14 software, was used to represent the genetic distance between the 11 genetic groups (Supplementary Fig. 5).

### Genetic analyses

Two types of genetic analyses were then carried out for each archaeological sample analyzed individually with the reference collection:

#### Structure analyses

We used a Bayesian model-based clustering method, implemented in the STRUCTURE software V2.3.4^35^, to identify distinct genetic groups in the reference collection (*T. cacao* or its wild relatives), and to establish the genotype membership proportion of each genetic group for each archaeological item using the same set of SNP markers. STRUCTURE was run under an admixture model, using a burn-in initial period of 100,000, a run length of 300,000 steps, and 10 independent runs for each sample where K values equalled from eight to twelve. Only archaeological samples with a minimum of 20 SNP markers were considered for these analyses.

The different genetic groups can be dissociated at different values of K, according to the number of SNP markers revealed in the set of aDNA sequences. We started to carry out the analyses with a maximum of K = 12 corresponding to the known structured modern populations. When the analyses with K = 12 did not allow to clearly distinguish the components of ancestry of the archaeological item, we continue the analyses, gradually decreasing the K values. Generally, we obtained this distinction with K values higher that K = 8. For each sample the higher K value allowing to clearly infer ancestry of archaeological samples to *T. cacao* genetic groups was chosen (Fig. 4, Supplementary Table 3, Supplementary Fig. 6).

When it was not possible to differentiate the genotype membership proportion between two given groups, due to their higher relatedness (Supplementary Fig. 5), as Nanay and Amelonado, and to the number of SNP used for the analysis, both possible attributions were reported in the Supplementary Table 3.

#### Genetic distances

With each set of SNP markers identified commonly for each archaeological sample and the reference collection, Nei’s genetic distances^74^, adapted to small effective size, were calculated between the ancient DNA sequences, selected as specific *T. cacao* or *Herrania* sequences, and the other 12 genetic groups (including the five wild *Theobroma* and *Herrania* accessions taken as a separate group) using GENETIX software V4.05.2^75^ (Supplementary Table 4).

### Supplementary Information

Supplementary Information.

## Data Availability

Raw sequence reads were deposited in the Sequence Read Archive (SRA) of the National Center for Biotechnology Information (NCBI) (BioProject: PRJNA1049643), and accessible at the following link: https://www.ncbi.nlm.nih.gov/sra/PRJNA1049643. The sequences specific to *T. cacao* (aDNA_T_cacao_sequences) and to *Herrania* (aDNA_Herrania_sequences) are available in the following websites: https://cocoa-genome-hub.southgreen.fr/sites/cocoa-genome-hub.southgreen.fr/files/download/aDNA_Herrania.fna.gz. https://cocoa-genome-hub.southgreen.fr/sites/cocoa-genome-hub.southgreen.fr/files/download/aDNA_T_cacao.fna.gz. The traits of human cultures associated with archaeological samples are reported in Supplementary Table 1. The other datasets are available from the corresponding author upon request.
