# Supplementary material for: A revisited history of cacao domestication in pre-Columbian times revealed by archaeogenomic approaches
Source: Sci Rep. 2024 Mar 7;14:2972. doi: 10.1038/s41598-024-53010-6 (PMC10920634; doi:10.1038/s41598-024-53010-6)
Supplement: Supplementary file 1 — Supplementary Information. [file 41598_2024_53010_MOESM1_ESM.docx]

**A revisited history of cacao domestication in pre-Columbian times revealed by archaeogenomic approaches**

**Lanaud et al.**

**SUPPLEMENTARY FILES**

1. **Supplementary notes**

I.1. Amazonian interactions

I.2. The Valdivia Culture

I.3. Monsú, Barlovento and Puerto Hormiga cultures

I.4. The Calima culture

I.5. Cacao presence in Central America

1. **Supplementary methods**

Library construction

1. **Supplementary figures**

Supplementary Figure 1. UPLC-MS/MS chromatograms of controls used for the methylxanthine analyses.

Supplementary Figure 2. Global distribution of methylxanthine values.

Supplementary Figure 3. Evidences of high level of fragmentation in ancient DNA

Supplementary Figure 4. Post mortem aDNA damages.

Supplementary Figure 5. Phylogenetic tree of *T. cacao* accessions representing the genetic distance between the 11 *T. cacao* genetic groups

Supplementary Figure 6. Bar plots of genetic structure analyzes.

Supplementary Figure 7. Bar plots of genetic structure analyzes from K=2 to K=12 for two Valdivia archaeological items.

Supplementary Figure 8. Visualization of metagenomic classification of ceramic residues sequences after mapping on the *T. cacao* genome.

1. **Supplementary tables**

Supplementary Table 1. Description of archaeological items analyzed for methylxanthine presence and/or *T. cacao* ancient DNA presence.

Supplementary Table 2. Detailed results of aDNA and methylxanthine analyzes (pg/sample) for the archaeological samples positive for *T. cacao/Herrania* aDNA and/or methylxanthine presence.

Supplementary Table 3. Genetic structure analyzes of archaeological item residues.

Supplementary Table 4. Nei genetic distances between ancient DNA sequences and modern *T. cacao* and wild relative genetic groups.

Supplementary Table 5. Reference collection.

Supplementary Table 6. Primer pairs, defined to show evidence of ancient DNA fragmentation.

1. **Supplementary references**

**I. Supplementary notes**

***I.1 Amazonian interactions***

The rapid spread of useful plants between Amazonia, the highlands and the Pacific coast cannot really be dated specifically because transmission is not always accounted for in terms of factual archaeological evidence. The case of the rapid spread of bananas/plantains in the norther part of South America is probably the best example. We know that bananas/plantains was first introduced to the New World with the arrival of Spanish in America between AD 1492 and 1500. Columbus and other Spanish and Portuguese brought bananas from Africa and Europe to feed the slave population that were part of the initial conquest. However, by 1542, when Orellana and his men “discovered” the Amazon, they found that plantains were harvested throughout the entire stretch of the Amazon Basin^1^. The Native populations found the use and merits of the fruit and its use reached the far ends of the continent far before the original introducers of the plant did so.

The same could be said of cacao and other plants that were originally used and domesticated in Amazonia thousands of years ago. It has been shown that *Theobroma cacao* was currently used by the Mayo-Chinchipe-Marañon culture in the upper Amazon by 5300 BP^18^ and there is evidence that the coeval Valdivia culture people of the Pacific coast were using related varieties at a similar time range, so the question is how were these plants crossing the Andes mountain range, and since when was this going on? The how part of the query can be explained by the geographic feature known as the Huancabamba Depression, which is the region where the Andean cordillera is at its lowest point in the continent. One can cross the Andes at altitudes of 2200 m or less and join the upper Amazon and the Pacific coast with relatively minimal effort. The low altitude region is not too far away from the Gulf of Guayaquil, facilitating contacts (direct or indirect) between both sides of the Andes. the Valdivian culture people could obtain different varieties of *Theobroma,* coca (*Erythroxlylum coca*), and other plants from their Amazonian counterparts without much effort^2^.

The known example of the Mayo-Chinchipe-Marañon culture can also be assumed for other upper Amazon people that thrived in the eastern flanks of the Andes and that were in close contact with their Andean and coastal counterparts of what is now Perú, Ecuador, and Colombia. In short, the early dispersal of the different *Theobroma* *cacao* varieties or *Theobroma* species can be traced genetically, but the direct archeological evidence of such contacts are currently hard to find, mostly through lack of archaeological research in the upper Amazon.

***I.2 The Valdivia Culture***

The Valdivia culture is the oldest of the cultures of the Formative Period in Ecuador, and it developed in the coastal areas of Ecuador. The Valdivia period was divided in eight phases dated by several authors. The eight-phase sequence first proposed^3^ spans from 3800 BC to 2000 BC. However, a recent reevaluation suggests a span from 3800-1450 BC^4^.

| Chronology | Phase |
| --- | --- |
| 1800 - 1450 BC | Phase VIII |
| 1950 - 1800 BC | Phase VII |
| 2100 - 1950 BC | Phase VI |
| 2250 - 2100 BC | Phase V |
| 2400 - 2250 BC | Phase IV |
| 2800 - 2400 BC | Phase III |
| 3300 - 2800 BC | Phase II |
| 3800 - 3300 BC | Phase I |

The Valdivia culture represents the beginning of the Neolithic Revolution in coastal Ecuador. It was a society based on agriculture and ceramic production, organized in village settlements with domestic and ceremonial areas. The Valdivia people showed a very good adaptation to the varied environments of the coastal lowlands (tropical dry forest, coastal strip, rolling savannah, mangroves, hydrographic basins), with agricultural production, elaboration of ceramic pieces for domestic and ritual use, and domestic and ceremonial burials^5^.

Archaeological similitude between Valdivia and Perú was reported for the last phases of Valdivia culture^6,7^.

***I.3. Monsú, Barlovento and Puerto Hormiga cultures***

Some of the sherds dated in this research are part of complex and diverse archaeological sites located on the Caribbean coast of Colombia. Some of these sites are dated from 4000 BC and are defined as Early Formative human settlements. It was proposed that probably before 4000 BC settlements called “malocas” existed in the region around Canal del Dique^8^; big communal houses that were inhabited by some nuclear families. These types of houses indicated a semi-sedentary living, the use of concentrated natural resources, and that probably some kind of itinerant horticulture and agriculture was already practiced. At the same time, semi-permanent or seasonal settlements also existed where the livelihood consisted of hunting and gathering, especially collecting enormous amounts of mollusks.

In these areas important food resources were concentrated and people formed low hills formed by an accumulation of soil, sand, and garbage (constituted by fragments of ceramics, bones, stones, and mollusk shells) called concheros. These concheros, formed over centuries and even millennia of occupations, were excavated for the very first time in the 1960´s^8-12^. They found that people used to live on these concheros in seasons where fishing was plentiful, and the amount of fruits and animals was abundant. These mounds were established in the fourth and third millennium BC in different parts of the Caribbean Coast of Colombia and constituted the first vestiges of ceramic cultures. The main archaeological site, which has so far produced the most ancient dates, is the mound of Monsú, located near the mouth of the Canal del Dique, near Cartagena de Indias. Many archaeological sites were found on its banks and Monsú is the westernmost of them. The people who lived there practiced a mixed economy, and the researchers suggested that it is very possible that the inhabitants of the oldest stratum already practiced a rudimentary form of agriculture, probably of cassava (*Manihot esculenta*) and other roots. They were gatherers of seeds and fruits of palms and collected turtles, crabs and mollusks; they exploited the resources of the sea, lagoons, estuaries, riparian forest and savannas.

The mound was divided into a cultural sequence of five great periods: Turbana, Monsú, Pangola, Macaví and Barlovento. In the research, they obtained a radiocarbon date for the Monsú period of 3350 BC.

Barlovento is the most recent period for Monsú mound, and it is also the name of an archaeological site excavated by Reichel-Dolmatoff, located northeast of Cartagena and part of the same ceramic complex. The Barlovento site is dated between 1500 and 1000 BC and the date for the Barlovento period in Monsú is 1300 BC. In the aforementioned period, hoes made of large shells (*Strombus gigas*) were found. Some of these tools were probably attached to an angled handle, while others seem to have been used with a long, thick vertical rod, used to loosen soil. These tools are testimonies of agriculture.

Another archaeological site of the Early Formative period is Puerto Hormiga^10^, close to Monsú on the Canal del Dique. Puerto Hormiga is a conchero located on low ground, a few meters above sea level. The site measures approximately 80 meters in diameter. The mound consists of a series of deposits of shells mixed with ceramic fragments, stone artifacts, and the bones of prey animals. Like the case of Monsú, the mound was occupied many times, as evidenced by many hearths and trampled surfaces. The structure of the mound indicates that, from the beginning, its inhabitants lived in a great circle where each family unit came to accumulate a small mound of waste that, at its base, began to overlap with the neighboring mounds thus forming a wide raised ring. In the different strata of shells, were found different styles of sherds from the beginning of the settlement^10^. The inhabitants of Puerto Hormiga were gatherers, whose subsistence was based mainly on fluvial resources (mollusks, fish, reptiles, etc.) and additionally on the collection of nuts and wild seeds.

The inhabitants of Puerto Hormiga seem to have been authentic gatherers, but their eating habits clearly included some amount of plant resources, and it is possible that they practiced some form or incipient horticulture with radiocarbon samples dated between 3100 BC to 2500 BC^10^.

The ways of life described of the farmers of Monsú and the hunters, fishermen, and perhaps incipient horticulturists of Puerto Hormiga, on their large trash heaps, continue on for a good part of the lowlands of the Caribbean Coast and developed for more than twenty centuries, without truly fundamental changes^10^.

***I.4. Calima culture***

The archaeological region called Calima designates the wide valley of the upper Calima river in the Eastern Cordillera of Colombia and its surrounding areas. It also encompasses the high regions of the towns of Yotoco, Vijes, Restrepo, Palmira and Cerrito close to the Cauca river. This area was inhabited for 9000 years, first by hunter-gatherers, and then by farmers, potters and goldsmiths. Archaeologists divided the history of this archaeological region into periods called: preceramic, Ilama, Yotoco-Malagana and Sonso.

The samples studied for cacao and methylxanthine presence belong to the Ilama period (1500 – 100 BC)^13^. The people who inhabited this area during this time established themselves in the Calima region by 1500 BC. The name of “Ilama” corresponds to the name of a village where an extensive archaeological zone has been found to the south of the municipality of Restrepo. The radiocarbon dates available^13^ show that this human group existed for a little more than a millennium. It has been suggested that this population could have arrived from the Pacific coast, taking advantage of the natural routes of the rivers that descend from the western slope of the Western Cordillera^13^.

Little is known about the social, political, and economic organization of the societies that lived in that period of time. Nevertheless, this period marks the beginning of pottery in that region. For the societies that inhabited this area, making pottery was a way to materially express the world around them. A great variety of human and animal forms, as well as moments of daily life, were artistically represented in the ceramics, suggesting the existence of social relations of gender, power and hierarchy. The ceramic vessels highlight the links between human beings and nature, the everyday world and the supernatural one. The physical appearance of people, their hairstyles and adornments, and their lifestyles, can be seen in these ceramics.

There is evidence of contact with other pre-Columbian societies, but the information is scarce because the main research of Ilama period and the Calima archaeological region has focused until now on goldsmith and pottery.

***I.5. Cacao presence in Central America***

Archaeological evidence has shown the economic, social and cultural importance^7^ of cacao in Central America. For instance, absorbed residues found on pottery belonging to the Mokaya people who lived at the site of Paso de la Amada, provided chemical evidence (methylxanthine) of cacao use by 1900 BC on the Pacific Coast of Chiapas in Mexico^7,10^. Around the same time (ca. 1800-1600 BC), cacao use is documented at the Olmec capital of San Lorenzo and the sacred bog site of El Manati, both located in Veracruz^12,7,10,11^.

Evidence of cacao use just outside the Maya area in northern Honduras at the site of Puerto Escondido in the Ulua Valley dates to 1400-110 BC. In the Maya area, it is found much later at the site of Colha located in northern Belize around 600 BC^5,6^. Exactly how cacao spread from the Mokaya to Olmec and from the Olmec to the Maya cultures is still being debated. Cacao may have been traded/exchanged between the Olmec and Maya along with jade, obsidian, and shell.

Our understanding of the presence of cacao in Lower Central America is limited; however, its occurrence archaeologically (presence of macrobotanical remains) may date to the Bagaces period (AD 300-800) where archaeologists have traced its use/distribution to the Greater Nicoya region of Nicaragua and Costa Rica^14^.

1. **Supplementary methods**

Libraries construction

Dual-indexed Illumina sequencing libraries were prepared using three main reactions^56^: blunt end-repair, adapter ligation, and nick fill-in reaction.

For the end-repair reaction, the following components were mixed in a 1.5 ml Eppendorf LoBind tube: 25 μl of sample DNA, 3 μl of NEBNext 10x End Repair Reaction Buffer, and 1.5 μl of NEBNext End Repair Enzyme Mix. The reaction was incubated for 15 minutes at 25°C and then purified using a MinElute spin column (MinElute PCR purification Kit, Qiagen). The purification was performed using 190 μl of modified PB binding buffer and 10 μl of sodium acetate^15^ and was centrifuged at 6,000 g. The column was washed with 750 μl of PE buffer and spun at 10,000 g, followed by an additional spin for 1 minute at 17,000 g. The DNA was eluted in 19 μl of EBT buffer (identical to QIAGEN’s EB buffer), with an incubation at 30°C for 5 minutes before collecting the DNA at 16,000 g.

For the ligation reaction, 1 μl of Illumina adapters at 5 μM were thoroughly mixed with the end-repaired DNA, followed by 8 μl of 5x NEBNext Quick Ligation Reaction Buffer and 1 μl of Quick T4 DNA Ligase. The reaction was incubated for 30 minutes at 22°C. The adapter-ligated DNA was purified using a Qiagen MinElute spin column, as before, with an elution volume of 21 μl.

The fill-in reaction was performed using 3 μl of 10x NEBNext Adapter Fill-in Reaction Buffer and 1 μl of Bst DNA Polymerase, Large Fragment, with an incubation at 37°C for 20 minutes followed by 80°C for 10 minutes. Before indexing the libraries by PCR using Pfu Turbo Cx HotStart DNA polymerase (Agilent), a qPCR in 15 μl was conducted to evaluate the minimum number of cycles needed to create an optimal library. Finally, the libraries were purified using magnetic beads (AMPure XP, Beckman, Coulter) at 1.8 x volume and then characterized by the 4200 TapeSation System (Agilent) to pool the libraries by equimolarity for sequencing by a NOVASEQ 6000 Illumina sequencer.

1. **Supplementary figures**

**Supplementary Figure 1**. **UPLC-MS/MS chromatograms of controls used for the methylxanthine analyzes.**

**A:** Positive control with a commercial solution of methylxanthine; **B**: join analysis of sample and methylxanthine solution, showing a unique spike; **C**: LCMS (liquid chromatography – mass spectrometry) blank (negative control), showing no contamination through LCMS; **D**: Ceramic samples analyzed individually (Chorrera ceramic items, representing: for theobromine, P57, an effigy vessel of a pregnant woman; for theophylline, P151, a whistling bottle; for caffeine, P137 a bottle); **E**: sample preparation blank (negative control) made with one sterile swab and showing no contamination during extraction protocol.

**Supplementary Figure 2**. **Global distribution of methylxanthine values.** Distributions of theobromine, caffeine and theophylline values among the 326 archaeological samples analyzed for methylxanthines, showing a clear break in the distributions after the values of 200 pg/sample, likely reflecting the limits of detection and/or the environmental background.

**
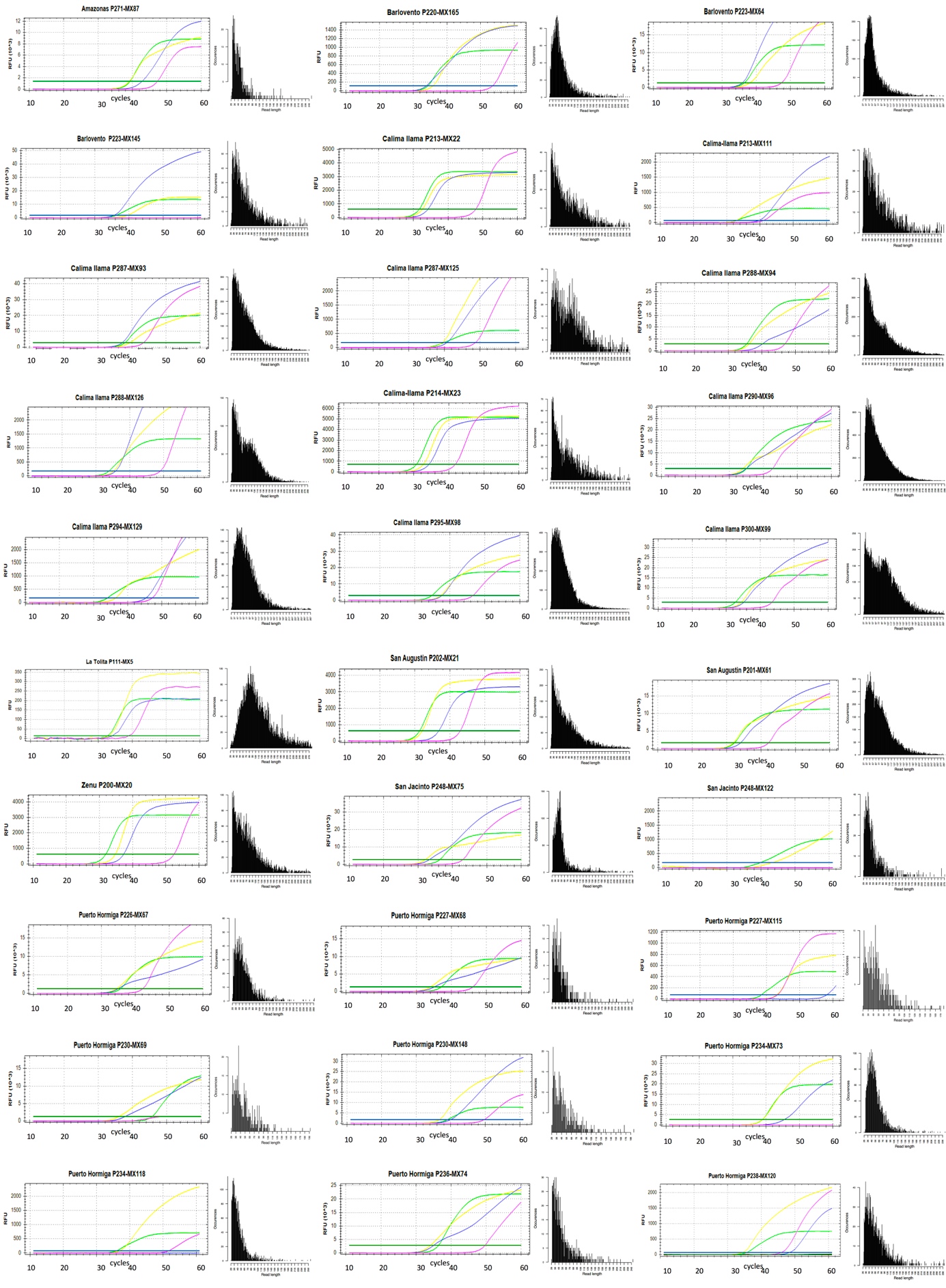
**

**
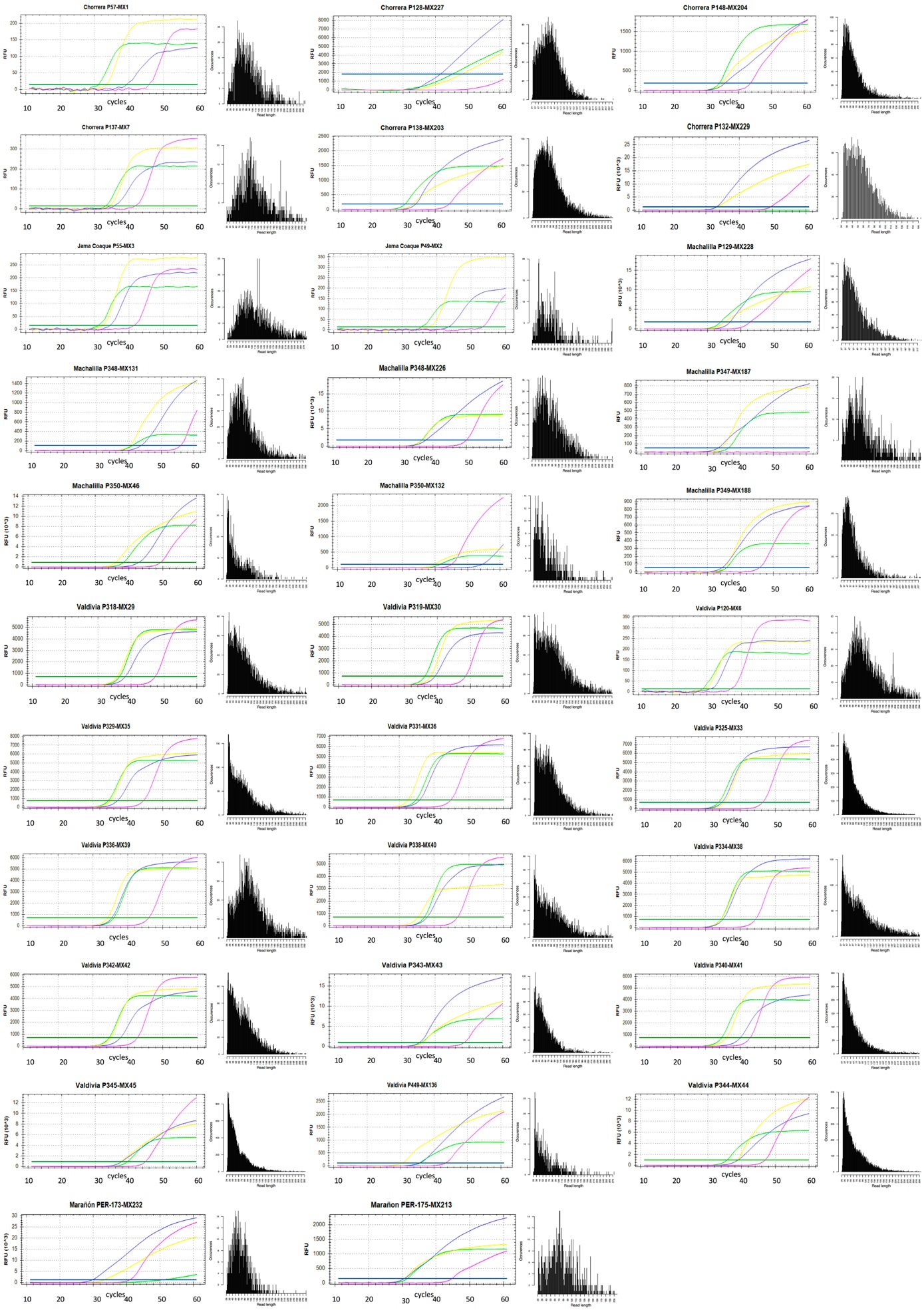
**

**
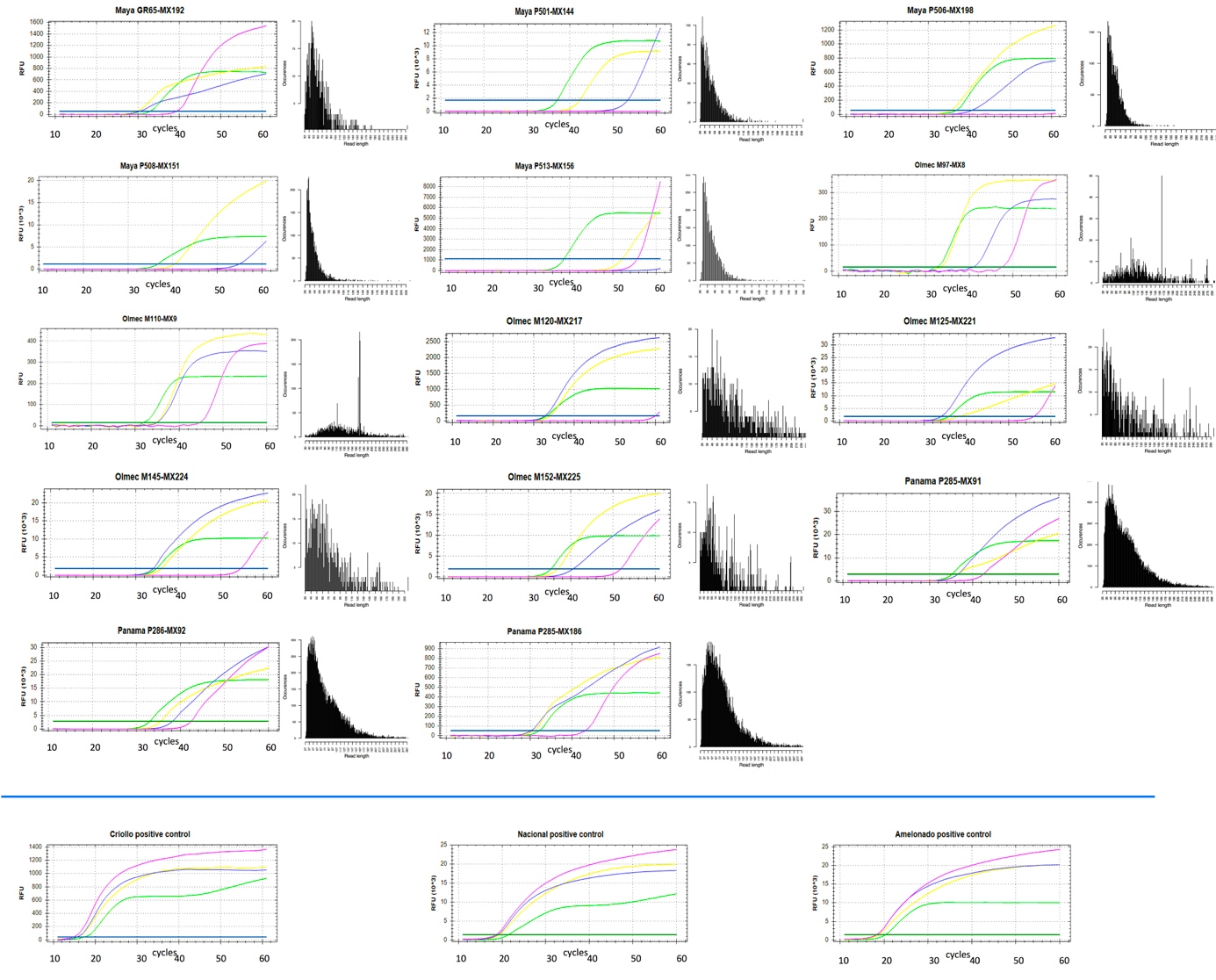
**

# Supplementary Figure 3. Evidences of high level of fragmentation in ancient DNA. Real-time PCR amplifications of aDNA extracts were carried out. Primers pairs corresponding to the amplification of increased lengths of mitochondrial “*Cytochrome C Oxidase* subunit 2” DNA fragments were used to compare their relative efficacy for amplifying aDNA extracts: (green) Cyto 66b: 66 bp; (yellow) Mito197: 197 bp; (blue) Mito 290: 290 bp; (purple) Mito 543: 543 bp.

# The relative fluorescence units (RFUs) is plotted against cycles number. A decreased PCR amplification intensity or no amplification can be observed with primers amplifying the longer aDNA fragment (543 bp), which is not observed in the three modern *T. cacao* positive controls: Criollo, Nacional and Amelonado samples. The read length distribution given by MapDamage^67^ is also reported for each of the aDNA samples. Among all samples analyzed, the extraction of 30 of them were duplicated and analyzed independently.

**
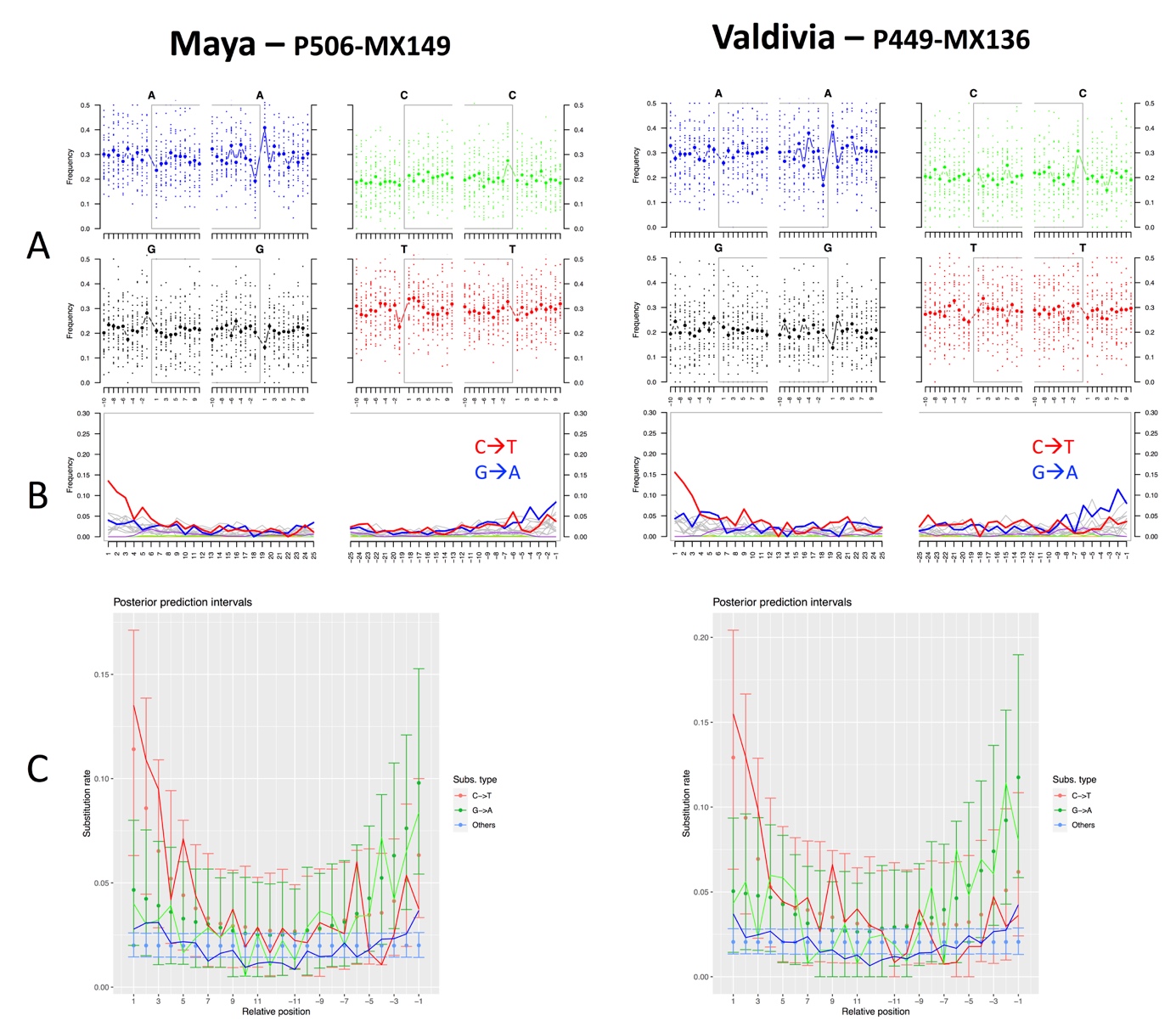
**

**Supplementary Figure 4.** **Post mortem aDNA damages**. MapDamage^66^ plots were observed in selected aDNA sequences obtained after mapping the assembled reads pairs from NOVASEQ 6000 in the *T. cacao* genome. Examples for a Maya sample (P506) and a Valdivia (P449) sample with respectively 1532 and 510 mapped sequences:

**A** - Base composition around the reads ends. The occurrence of purine (A and G) deamination can lead to DNA fragmentation, and an enrichment of purines can be observed around the reads aDNA ends.

**B** and **C** - Cytosine deamination damage leading to mis-incorporation pattern at the ends of aDNA sequences. Frequency of C->T and G->A substitutions are reported according to the base position along the aDNA fragments. They occur more frequently at 5-prime ends (C->T) and at 3-prime ends (G->A) of aDNA fragments.


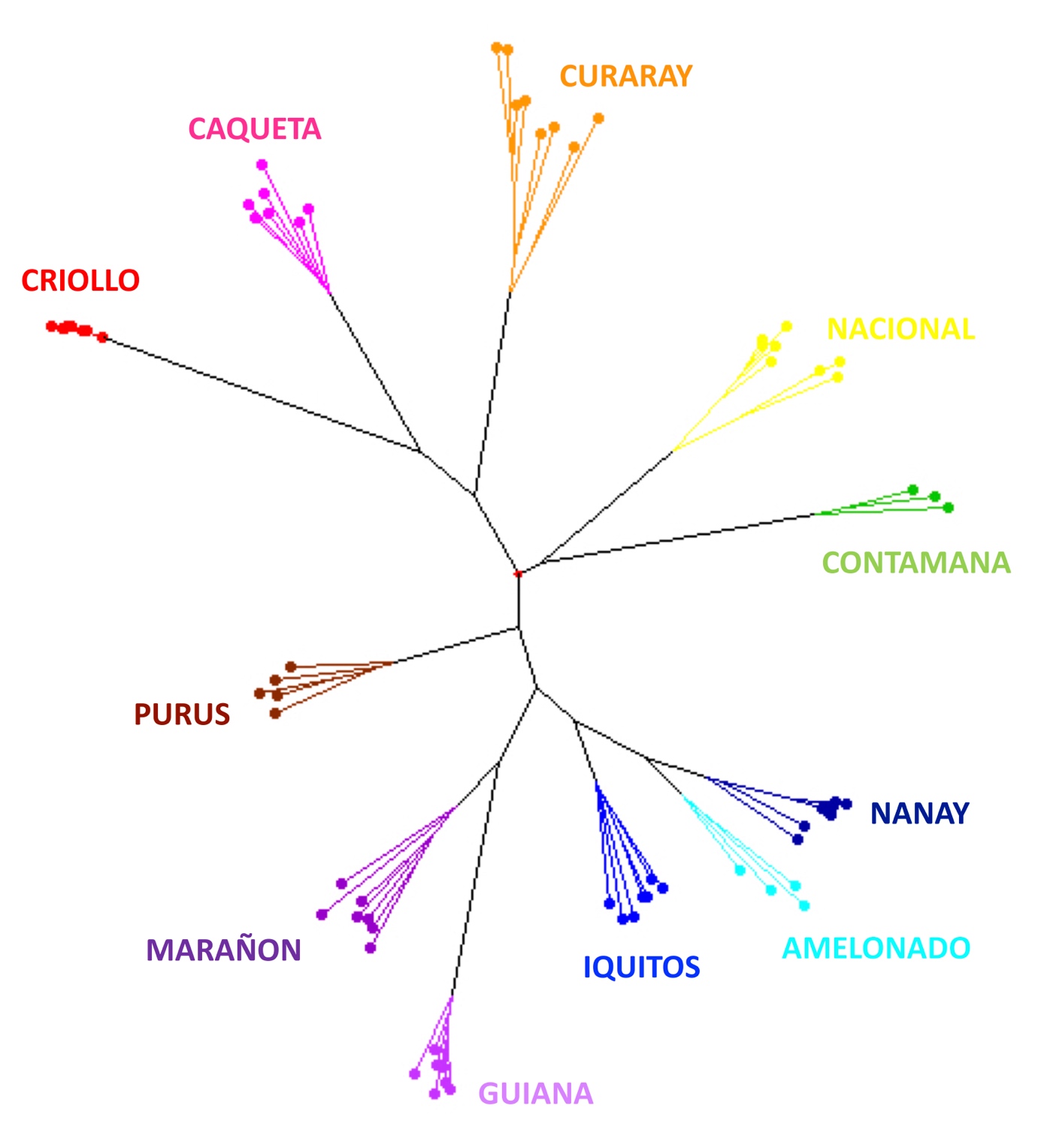


**Supplementary Figure 5. Phylogenetic tree of *T. cacao* accessions representing the genetic distance between the 11 *T. cacao* genetic groups.** The phylogenic tree was constructed with the Darwin software^72^, with the 76 *T. cacao* accessions, belonging to the 11 genetic groups^4^, genotyped by 460 SNP markers and used as comparison to evaluate the genetic structure and distance of archaeological cacao aDNA to the genetic groups in this study. The total length of the branches between two given genetic groups is proportional to the genetic distance between them.

**Supplementary Figure 6. Bar plots of genetic structure analyses.** The cluster membership proportion generated by Structure software^35^ is reported for a collection of 76 modern accessions representing the diversity of *T. cacao* species, a group of 5 accessions of wild relatives (*Herrania nitida, T. bicolor, T. grandiflorum and T. speciosum*), and the aDNA mixture extracted from the ceramic residues. Archaeological name and number of SNP, in parentheses, are mentioned at the left of each line. Each individual is represented by a single vertical line and clusters, generally corresponding to the several *T. cacao* and wild genetic groups, are represented by a different color given by Structure software. Each aDNA extract was analyzed independently with the collection of modern accessions (see also Supplementary Table 3 for detailed values). The average estimated cluster membership of each aDNA extract is visualized, at the end of each plot, by a single vertical line with colored segment lengths proportional to each of the K inferred clusters.

**Supplementary Figure 7. Bar plots of genetic structure analyses from K=2 to K=12 for two Valdivia archaeological items.** The cluster membership proportion generated by Structure software^35^ is reported from K=2 to K=12, showing a consistent ancestry to Amelonado group, for P334, and to Criollo and Nacional groups for P340 for all values of K.


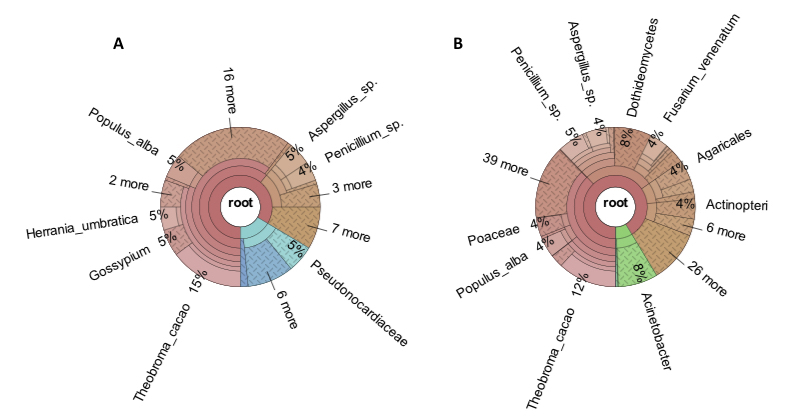


**Supplementary Fig. 8. Visualization of metagenomic classification of ceramic residues sequences after mapping on the *T. cacao* genome.**

The Krona schemes^65^ allow the visualization of relative abundances of taxonomical groups among meta-genomic data obtained after mapping of whole sequencing data on the *T. cacao* genome, for two archaeological samples. **A**: P230 Puerto Hormiga archaeological item, out of 128 sequences mapped in the *T. cacao* genome, 19 sequences (15%) were more similar (first hit) to *T. cacao* sequences, and six (5%) were more similar to *Herrania* sequences, after BLAST on international NCBI NT database. **B**: P120 Valdivia archaeological item, out of 6197 sequences mapped in the *T. cacao* genome, 737 sequences (12%) were more similar to *T. cacao* sequences after BLAST on international NCBI NT database.

1. **Supplementary tables**

**Supplementary Table 1. Description of archaeological items analyzed for methylxanthine presence and/or *T. cacao* ancient DNA presence.**

MAAC: Museo Antropologico y de Arte Contemporaneo ; MNE: Museo Nacional de Ecuador; MNC: Museo Nacional de Colombia ; ICANH: Instituto Colombiano de Antropología e Historia ; KSU: Kennesaw State University; UNAM (Universidad Nacional Autónoma de México);

AR: archaeological reserve

(AE)1: Archaeological excavation^16^

(AE)2: Archaeological excavation^9^

(AE)3: Archaeological excavation^10^

*: samples positive for theobromine or theophylline (column J) and positive for *T. cacao* (column K); TC: targeted capture; WGS: whole genome sequencing

**Supplementary Table 2. Detailed results of aDNA and methylxanthine analyses (pg/sample) for the archaeological samples positive for *T. cacao/Herrania* aDNA and/or methylxanthine presence.** All values of methylxanthine < 700 pg/sample, taken as the threshold to declare a positive sample, were reported as “0” in this table. NA: Not Analyzed.

**Supplementary Table 3. Genetic structure analyses of archaeological item residues.**

Genotype membership proportions of *T. cacao* genetic groups and wild relatives were estimated for each archaeological item using a Bayesian model-based clustering method, implemented in the STRUCTURE software V2.3.4.^35^. Each archaeological item was analyzed individually with the reference collection, with a set of SNP markers identified in its ancient DNA sequences and common with the reference collection. When it was not possible to differentiate the genotype member proportion between two given genetic groups, it was reported in the table for both genetic groups, with the mention “or”.

**Supplementary Table 4. Nei genetic distances between ancient DNA sequences and modern *T. cacao* and wild relative genetic groups.** Genetic distances were calculated for each archaeological sample with the GENETIX software V4.05.2^75^, and the Nei 78 distances^74^ adapted to small effective size. Twelve genetic groups were considered: the eleven genetic groups recently reported^4^ and a wild relative group including *T. bicolor*, *T. grandiflorum*, *T. speciosum* and *Herrania nitida*.

**Supplementary Table 5. Reference collection.** Origin of modern accessions used to establish, for each archaeological sample, the genotype membership proportion and the Nei genetic distance to the several *T. cacao* genetic groups. This collection contains representatives of the eleven groups identified^4^ (76 accessions) and representatives of three relative *Theobroma* species and one *Herrania* species.

INTA: Nicaraguan Institute of Agricultural Technology (Nicaragua); CIC: Centro de Investigacion Caribia (Colombia); CRC: Cocoa Research Center (Trinidad and Tobago); CATIE: Centro Agronómico Tropical de Investigación y Enseñanza (Costa Rica); INIAP-EETP: Instituto Nacional de Investigaciones Agropecuarias (Ecuador); INIAP-ECCA: Instituto Nacional de Investigaciones Agropecuarias (Ecuador); CIRAD: Centre de Coopération Internationale en Recherche Agronomique pour le Développement (France).

**Supplementary Table 6. Primer pairs, defined to show evidence of ancient DNA fragmentation.** Four Primers pairs defined in the mitochondrial *Cytochrome oxidase* gene and having a similar PCR efficiency were used to amplify DNA fragments differing for their size (66 bp, 197 bp, 290 bp, 543 bp). These primers allowed to check the decreased amplification level of ancient DNA with primers amplifying longer DNA fragments, which is a characteristic of ancient DNA.

**V. Supplementary references**

1) Carvajal, G. Relación del nuevo descubrimiento del famoso Río Grande que descubrió por muy gran ventura el Capitán Francisco de Orellana. Transcripciones de Fernández de Oviedo y Dn. Toribio Medina y estudio crítico del descubrimiento, Repositorio Institucional de la Universidad de Cuenca, biblioteca Amazonas, vol **1**, <http://dspace.ucuenca.edu.ec/handle/123456789/27556>. (1942).

2) Marcos, J.G. Los 10.000 Años del Antiguo Ecuador. La Historia de sus Pueblos a través de su Arte y Ciencia. Museo Antropológico y Arte Contemporáneo, Guayaquil. 104 p (2006).

3) Hill, B. D. A New Chronology of the Valdivia Complex from the coastal Zone of Guayas Province, Ecuador. *Ñawpa Pacha* **10-12**:1-32. (1972-74).

4) Lunniss, R. M., Zeidler, J. A., and Ortiz Aguilú, J. J. La transición Arcaico Tardío - Valdivia: una reevaluación en base a evidencias se los sitios Salango y Valdivia. In *Valdivia, un sociedad neolítica: nuevos aportes a su conocimiento*, edited by Mary B. Jadán Veriñez, pp. 120-158. Ediciones UTM, Manta, Ecuador. (2021).

5) García Caputi, M. La Figurina Valdivia: El Ícono americano de la Revolución Neolítica” Exhibit and catalogue, MAAC. Impresión Archivo Histórico del Guayas. Guayaquil, Ecuador. (2019).

6) Estrada, E. Correlaciones entre la arqueología de la costa del Ecuador y Perú. *Humanitas*, Editorial Universitaria, pp. 31-61. Équateur. (1962).

7) Staller, J. E. The Jelí Phase Complex at La Emerenciana, A Late Valdivia Site in Southern El Oro Province, Ecuador. *Andean Past* **6**:117-174. (2000).

8) Reichel-Dolmatoff, G. Arqueología de Colombia: un texto introductorio. Bogotá: Banco de la República. (1997)

9) Reichel-Dolmatoff, G., & Reichel-Dolmatoff, A. Momíl, excavaciones en el Sinú. Revista Colombiana De Antropología, 5, 111–333. <https://doi.org/10.22380/2539472X.1800> . (1956).

10) Reichel-Dolmatoff, G. Puerto Hormiga: un complejo prehistórico marginal de Colombia. Bogotá: *Revista Colombiana De Antropología*, **10**, 349–354. (1961).

11) Reichel-Dolmatoff, G., and Dussán de Reichel, A. Investigaciones arqueológicas en la Costa Pacífica de Colombia; II: Una secuencia cultural del bajo río San Juan. *Revista Colombiana de Antropología* **11**:9-70. (1962).

12) Reichel-Dolmatoff, G. *Colombia* Vol 44, Ancient Peoples and Places. London: Thames & Hudson. 182 p (1965).

13) Cardale de Schrimpff, M., Bray, W., & Herrera, L. Reconstruyendo el pasado en Calima. Resultados recientes. *Boletín Museo Del Oro*, **24**, 3–33. (1989).

14) Steinbrenner, L. Cacao in Greater Nicoya: Ethnohistory and a Unique Tradition. In Chocolate in Mesoamerica: A Cultural History of Cacao, edited by Cameron McNeil, pp. 253-270. University Press of Florida, Gainesville, FL. (2006).

15) Allentoft, M., Sikora, M., Sjögren, KG. et al. Population genomics of Bronze Age Eurasia. *Nature***522**, 167–172 <https://doi.org/10.1038/nature14507>.(2015).

16) Herrera, L., Bray, W., & McEwan, C. Datos sobre la arqueología de Araracuara. *Revista Colombiana De Antropología*, **23**, 185 - 251. <https://doi.org/10.22380/2539472X.1740> (1981).
